# Large lattice distortions and size-dependent bandgap modulation in epitaxial halide perovskite nanowires

**DOI:** 10.1038/s41467-020-14365-2

**Published:** 2020-01-24

**Authors:** Eitan Oksenberg, Aboma Merdasa, Lothar Houben, Ifat Kaplan-Ashiri, Amnon Rothman, Ivan G. Scheblykin, Eva L. Unger, Ernesto Joselevich

**Affiliations:** 1 Department of Materials and Interfaces Weizmann Institute of Science, Rehovot, 76100 Israel; 2 Helmholtz-Zentrum Berlin GmbH, Young Investigator Group Hybrid Materials Formation and Scaling, Albert Einstein Straße 16, Berlin, 12489 Germany; 3 Chemical Research Support, Weizmann Institute of Science, Rehovot, 76100 Israel; 4 Chemical Physics and Nano Lund, Lund University, Box 124, , Lund, 22100 Sweden

**Keywords:** Materials for energy and catalysis, Nanoscale materials, Optical materials and structures

## Abstract

Metal-halide perovskites have been shown to be remarkable and promising optoelectronic materials. However, despite ongoing research from multiple perspectives, some fundamental questions regarding their optoelectronic properties remain controversial. One reason is the high-variance of data collected from, often unstable, polycrystalline thin films. Here we use ordered arrays of stable, single-crystal cesium lead bromide (CsPbBr_3_) nanowires grown by surface-guided chemical vapor deposition to study fundamental properties of these semiconductors in a one-dimensional model system. Specifically, we uncover the origin of an unusually large size-dependent luminescence emission spectral blue-shift. Using multiple spatially resolved spectroscopy techniques, we establish that bandgap modulation causes the emission shift, and by correlation with state-of-the-art electron microscopy methods, we reveal its origin in substantial and uniform lattice rotations due to heteroepitaxial strain and lattice relaxation. Understanding strain and its effect on the optoelectronic properties of these dynamic materials, from the atomic scale up, is essential to evaluate their performance limits and fundamentals of charge carrier dynamics.

## Abstract (web summary)

Metal-halide perovskites are promising photovoltaic materials, but fundamental questions remain open due to their structural complexity. Here the authors show, by correlated microscopy and spectroscopy methods, that epitaxially induced lattice distortions drive a size dependent modulation of the bandgap in a homogeneous nanowire system.

## Introduction

Metal-halide perovskites (MHPs) have revolutionized the photovoltaics (PV) field owing to a rare combination of simple, low-cost, solution-based fabrication processes, and exceptional optoelectronic properties^1^. They encompass efficient charge generation, long carrier lifetimes and large diffusion lengths along with often low non-radiative trap densities due to high defect tolerance, all of which are favorable traits for efficient photovoltaic applications^2–7^. By virtue of large spin–orbit coupling and excellent absorption, optical gain and emission properties, MHPs thin films, bulk single-crystals and nanostructures were also investigated as building blocks for light-emitting diodes, photodetectors, lasers, and spintronic devices^5,8–12^. Despite the outstanding and ongoing research done on MHPs from multiple perspectives and disciplines, some fundamental questions still remain unanswered or controversial^13^. One reason for that is the structural complexity of polycrystalline thin films of hybrid organic–inorganic MHPs and their high sensitivity to humidity, temperature, light irradiation, etc. Adding to that, the optical properties of polycrystalline samples can be expected to vary locally but this variation is often spatially averaged out when measuring with conventional spectroscopic methods. These drawbacks can prove detrimental when searching for experimental evidence to support hypotheses for complex processes such as carrier transport, ion migration, recombination pathways, and spin properties^11,14–16^. Even when characterizing the crystal structure, lattice strain and the electronic band structure, the polycrystallinity of perovskite thin films samples adds degrees of complexity and uncertainty. Therefore, it would be highly beneficial to develop and explore more stable, well-defined and simple model systems, such as one-dimensional nanowires in order to probe local material properties and avoid spatial averaging over a variety of crystal configurations. Such model systems should allow an accurate separation of intrinsic properties and extrinsic effects, eventually leading to a better understanding of complex processes in MHPs.

Nanowires of MHPs provide a simplified one-dimensional system that can be used to investigate and model various processes. They have been explored as potential building blocks for various applications and have exhibited interesting properties including low threshold lasing and polarity-dependent photodetection^9,17–21^. Recently, few groups have reported horizontal growth of CsPb*X*_3_ (X=Cl, Br, I) microwires and nanowires on mica^22,23^, amorphous SiO_2_^24^, and sapphire^20^. Our group has reported the horizontal and aligned growth of cesium lead bromide (CsPbBr_3_) nanowires that form arrays with 6-fold and 2-fold symmetries, which reflect the symmetry or morphology of their sapphire substrates^8^. As with standard semiconductors, the assembly of nanowires into ordered arrays facilitated their integration into functional devices and, from a fundamental research standpoint, enables the parallel investigation of multiple highly homogenous nanowires^25–32^. Surface-guided horizontal CsPbBr_3_ nanowires constitute an advantageous system for research and applications. The all-inorganic CsPbBr_3_ is more stable than hybrid organic-inorganic MHPs, but exhibits similar properties including a similar band structure^17,33,34^. The nanowires are quasi-one-dimensional single-crystals with a uniform crystallographic orientation, and the ordered nanowire-array can be readily integrated into devices without special fabrication steps, enabling electronic and optoelectronic research and applications.

While characterizing the surface-guided CsPbBr_3_ nanowires, we observed a size-dependent photoluminescence spectral blue-shift well beyond the quantum confinement regime^8^. We found that as the height of the nanowire decreases from ~1.5 μm to ~45 nm, the photoluminescence (PL) emission peak blue-shifts from ~530 nm to ~510 nm, respectively. Such a size-dependent emission shift has been reported only a few times in the literature, but was observed for both methylammonium lead iodide (MAPbI_3_) and CsPbBr_3_ and for different material structures, including platelets, nanowires, and thin films^8,35–37^. For MAPbI_3_ thin films, D’Innocenzo et al. reported an emission blue-shift from ~775 nm to ~760 nm as the average crystallite size decreases from 10 μm to ~200 nm. They related the shift to a possible distortion in the Pb–I bond^35^. For MAPbI_3_ micro-platelets, Li et al. reported an emission blue-shift in both the tetragonal and orthorhombic phase as the thickness of the platelets decreases from 150 nm to 17 nm. They state that the origin of their observation is still unclear but might be due to a surface effect^36^. Wang et al. reported an emission blue-shift from ~530 to ~516 nm with decreasing thickness of CsPbBr_3_ platelets and suggested that this stems from strain that accumulates due to nontrivial strength of the van der Waals epitaxial interaction between the platelets and the mica substrate^37^. The recurrence of this phenomenon in different MHPs and different structures implies that this is a general feature that probably exists in many other MHPs systems. In polycrystalline thin films, domain size and strain-induced fluctuations that likely occur, were seemingly overlooked for various reasons, including the intrinsic inhomogeneity of perovskite thin films samples. To date, the origin of the emission spectral shift has not been determined^8^.

Here, we exploit the advantages of a stable and homogeneous nanowire system to investigate the size-dependent emission shift and to uncover its origin. We combine optical- and electron- microscopy and spectroscopy along with atomic force microscope (AFM) measurements to investigate ensembles of horizontal CsPbBr_3_ nanowires with different heights and single, tapered nanowires with a height gradient along their growth axis. The height-dependent luminescence emission blue-shift is observed in PL and, in higher spatial resolution, using scanning electron microscopy-cathodoluminescence (SEM-CL). Using PL-excitation microscopy (PLExMic)^38^, we demonstrate that the emission blue-shift is a result of a bandgap modulation rather than an optical phenomenon. We trace the origin of the bandgap modulation down to the atomic structure using state-of-the-art scanning transmission electron microscopy (STEM) and strain analysis. Employing nanobeam scanning electron diffraction (SED) and geometric phase analysis (GPA), we find a large and uniform lattice rotation, which relates to octahedral tilting. We discuss the origin of the lattice distortions and conclude that they stem from heteroepitaxial mismatch, accentuated by a significant difference between the thermal expansion coefficients of the sapphire substrate and the CsPbBr_3_ surface-guided nanowires. We identify the unusually large lattice rotation as the major contributor to the observed height-dependent bandgap modulation. These findings reveal the unusual structural lattice response of MHPs to heteroepitaxy-induced stain and underscore its impact on their optoelectronic properties. In accordance with recent reports that highlight the impact of strain on the optoelectronic properties and stability of polycrystalline MHPs thin films^39–46^, our observations of large lattice distortions at the atomic scale, could thus greatly contribute to better understanding MHPs and pushing them towards their performance limits.

## Results

### Surface-guided CsPbBr_3_ nanowires

**Fig. 1:**
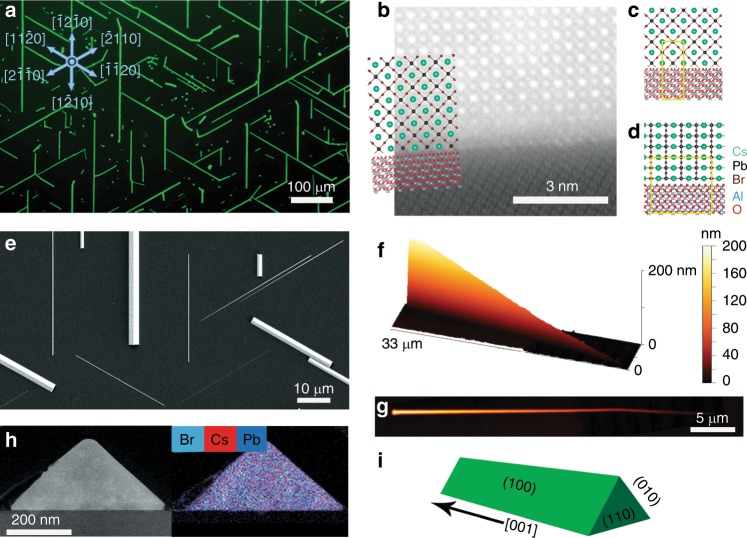
Surface-guided epitaxial CsPbBr_3_ nanowires on C-plane sapphire. (**a**) An optical photoluminescence image of surface-guided nanowires and microwires emitting green luminescence during wide-field excitation with a blue (405 nm laser). The growth directions of the nanowires on the sapphire substrate are marked with blue indices and vectors. (**b**) Cross-sectional atomic-resolution STEM HAADF image taken in the $[11\bar 20]$ viewing direction of the sapphire substrate. The atomistic model overlay shows the atomic positions that are associated with the bright dots in the HAADF image. Detailed view of the atomic model (**c**) across and (**d**) along the growth axis of the nanowires. The yellow rectangles mark the repeat period (see also Supplementary Fig. 1 for a top-view of the epitaxial relations). **e** SEM image of nanowires with highly varying heights. (**f**–**g**) A typical tapered nanowire with a height gradient is displayed in 3D (**f**) and 2D (**g**) AFM images. (**h**) TEM image and EDS elemental map of a typical triangular cross-section. (**i**) Illustration of the shape, facets and growth direction of CsPbBr_3_ surface-guided nanowires.

The vapor-phase growth of the surface-guided CsPbBr_3_ nanowires on sapphire was carried out in a three-zone tube furnace as described in the Methods section^8^. For simplicity, we choose to focus on CsPbBr_3_ nanowires that grow on a flat substrate, specifically flat C-plane sapphire. The uncatalyzed growth produces almost exclusively horizontally aligned nanowires and microwires that grow along the six isoperiodic A $\pm <11\bar 20>$ directions of the sapphire substrate, reflecting its symmetry (Fig. 1a and Supplementary Fig. 1). The typical length of the nanowires is a few tens of microns. The nanowires have a triangular cross-section, such that the height of a typical nanowire is about half its width. While some control over the lengths and heights of the nanowires can be attained with careful optimization of the growth time and conditions, their heights can vary considerably between 10 nm and a few microns. The epitaxial alignment and the height variance can be appreciated in Fig. 1e. Tapered nanowires with a height gradient along their growth axis can also be obtained. As can be seen with AFM 3D and 2D imaging in Fig. 1f, g, the height of this nanowire decreases from ~200 nm to ~10 nm in an almost linear manner throughout the 33 μm length of the nanowire (Supplementary Fig. 2), with a gradient of approximately 6 nm every micron. This height gradient can be used to effectively sample, in a continuous manner, the height-dependent optical and structural properties over a wide range of heights within a single nanowire using spatially resolved methods.

In order to enable TEM imaging and analysis, thin cross-sectional lamellae were cut using a focused-ion beam microscope (FIB). Special care was taken to ensure that beam irradiation doses and exposure times were minimized in order to prevent beam-induced damage or structural distortion that could otherwise effect the pristine lattice properties during the imaging and analysis (for details see Methods). A distinct isosceles right triangle cross-section shape of the CsPbBr_3_ nanowires is clearly observed in Fig. 1h, and energy dispersive X-ray spectroscopy (EDS) mapping reveals that the Cs, Pb, and Br elements, with the expected ~1:1:3 atomic ratio, are distributed uniformly throughout the cross-section (see Supplementary Fig. 3 for details). The nanowires grow in a cubic phase as triangular prisms with a [001] growth axis. The (110) plane is parallel to the (0001) plane of the sapphire and the (100) and (010) facets are exposed to the gas phase. The shape, facets and growth direction of CsPbBr_3_ surface-guided nanowires are illustrated in Fig. 1i.

Overlaying the bulk atomistic models of sapphire and CsPbBr_3_ on an atomic-resolution STEM high-angle annular dark-field (HAADF) image of a typical nanowire cross-section indicates a commensurate epitaxial growth (Fig. 1b–d). The positions of the overlaid atoms, using bulk lattice parameters as reference, are associated with the bright dots in the HAADF image. Although we could not atomically resolve the entire sapphire-CsPbBr_3_ interface, the STEM image together with overlaid atoms in the $[11\bar 20]$ viewing direction of the sapphire substrate, provide a detailed view of the atomic model and reveal the epitaxial relations as the CsPbBr_3_ (110) plane grows on the sapphire (0001) plane. Coinciding vectors of the repeating lattice period are highlighted with yellow rectangles as a $[1\bar 10]$ CsPbBr_3_ vector matches a $[\bar 1100]$ vector in the sapphire lattice in a 1:1 lattice ratio, giving a mismatch of +0.7% (Fig. 1c). Based on this epitaxial relation, a commensurate lattice can be assumed to form along the nanowire, in the orthogonal $[\bar 1100]$ viewing direction (Fig. 1d). Here, a [001] CsPbBr_3_ vector coincides with a $[11\bar 20]$ sapphire vector in a 4:5 lattice ratio, and the mismatch is −1.3%. In addition to TEM analyses, the existence of the CsPbBr_3_ cubic phase at room temperature was confirmed by XRD measurements done on as-grown surface-guided CsPbBr_3_ nanowires^8^.

### Size-dependent emission spectral shift

The height-dependent emission blue-shift can be investigated both on the ensemble and the individual nanowire level. Either an ensemble of non-tapered nanowires with varying heights, or a single tapered nanowire with a height gradient along its growth axis. We use two microscopy-based spectroscopic methods to probe, in a spatially resolved manner, the emission properties of the surface-guided nanowires: an optical excitation with varied excitation energies in PL measurements, yielding spatially resolved PL-excitation and emission spectra, and a high-energy electron-beam excitation yielding high-spatial resolution SEM-CL measurements.

**Fig. 2:**
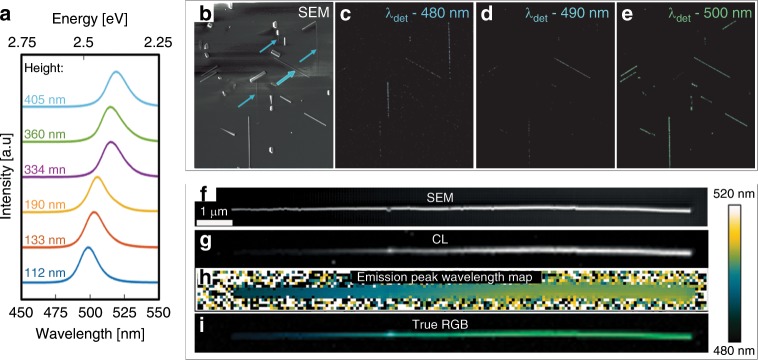
Emission spectroscopy of surface-guided epitaxial CsPbBr_3_ nanowires on C-plane sapphire. (**a**) PL spectra of nanowires with different heights (indicated in the figure) excited at 458 nm. SEM-CL monochromatic images of an ensemble of nanowires with varying heights: (**b**) SEM image and (**c**–**e**) CL monochromatic images generated by collecting the emitted light specifically at 480, 490, and 500 nm, displaying the emission wavelength distribution of the nanowires. Spatially resolved CL spectroscopy on a single tapered nanowire: (**f**) SEM image, (**g**) CL map, (**h**) the extracted emission peak wavelength map with its color-scale to its right, and (**i**) a true RGB color map calculated from the fitted emission peak displaying the varying emission wavelength, from green to blue, with the diminishing height of the tapered nanowire.

PL emission spectra were measured using a home-built PL microscopy set-up using a 458 nm continuous-wave (CW) excitation and a long-pass filter set to capture the entire emission spectra (for details see Supplementary Fig. 4 and Methods). As opposed to point-scanning confocal acquisition of an image, we employ wide-field excitation, which excites PL throughout the whole nanowire or ensemble of nanowires. We can thus acquire emission spectra simultaneously for every point along the nanowire with a spatial resolution of ~500 nm and a spectral resolution of 2.1 nm. In Fig. 2a, the PL spectra of an ensemble of CsPbBr_3_ nanowires with different heights are shown. The spectra correspond to the emission averaged across a region at the center of the probed nanowire.

Considering the calculated 7.5 nm Bohr-exciton radius for CsPbBr_3_^47^, the dimensions of the investigated nanowires are well beyond the quantum confinement regime. Still, with decreasing nanowire heights from ~400 to ~100 nm, a significant emission blue-shift from ~525 to ~490 nm (~2.36 eV to ~2.53 eV) is observed while the full width at half-maximum (FWHM) remains fairly constant at ~0.12 eV (Fig. 2a and Supplementary Fig. 5). These measurements are not directly compared with bulk CsPbBr_3_ PL due to inherent differences between the free-standing orthorhombic bulk CsPbBr_3_ system and these surface-bound cubic nanowires. We did, however, examine the emission behavior of horizontal CsPbBr_3_ nanowires with different heights that were grown on an amorphous SiO_2_ substrate and did not find an apparent height-dependent trend (Supplementary Fig. 6). The fact that the height-dependent spectral shift is observed for epitaxial CsPbBr_3_ nanowires grown on single-crystal sapphire but not when the nanowires are grown on an amorphous SiO_2_ substrate, suggests that the emission blue-shift is related to the interaction between the nanowires and substrate. This comparison strongly supports the attribution of the size-dependent spectral shift to heteroepitaxial strain, as we shall further elaborate with additional data.

To acquire spectra with a higher spatial resolution, we used a Gatan MonoCL Elite system that is installed in a Zeiss GeminiSEM 500, high-resolution SEM to conduct SEM-CL measurements. First, an SEM image and three CL images in a monochromatic mode were generated by collecting the emitted light at specific wavelengths using a high sensitivity photomultiplier tube (Fig. 2(b–e)). The CL monochromatic images where taken at 480, 490, and 500 nm. The ensemble of nanowires can thus be distinguished in the images according to their corresponding emission wavelengths. The 480 nm image (Fig. 2c) displays only two nanowires with heights of 20–80 nm that emit at ~480 nm, whereas many nanowires, as well as other nanostructures, with heights between 100 and 300 nm emit at ~500 nm and therefore are seen in the 500 nm image (Fig. 2e). These monochromatic images reveal that electron-beam excitation generates a similar distinction between the emission of small and large nanowires as observed with the optical excitation.

Next, we harnessed the high-spatial resolution of SEM-CL in order to follow the emission along a single tapered nanowire with a height gradient (Fig. 2(f–i)). The examined tapered nanowire is ~16 μm long (Fig. 2f), and contains two tapering regimes. Starting from the thick end, its height starts at the maximum value of 260 nm and effectively remains unchanged for the first ~5 μm, followed by a rapid decrease towards its thinner edge down to ~80 nm. The CL map of the nanowire is generated by exciting the nanowire with the electron beam and directing the emitted light to a monochromator and a CCD in parallel. A CL spectrum (450–520 nm) is generated for each 100 nm^2^ pixel area and the integrated intensity of the emission peak for the pixels generates the gray-scale contrast of the map in Fig. 2g. A Gaussian is then fitted to the emission peak at each pixel position, and the extracted wavelength of the emission peak is displayed using a false color table ranging between 480 and 520 nm. This generates the emission peak wavelength map (Fig. 2h). Next, the fitted Gaussian is used to determine the real RGB components of the emission and a true-color RGB image is calculated (Fig. 2i). From these measurements, the green-to-blue trend with decreasing height of the nanowire is clearly observed as the emission peak wavelength shifts from 504 nm (~2.46 eV) at the thick edge to 490 nm (~2.53 eV) close to the thin edge. Additional information including the goodness of the fit and the FWHM that correlates with the thickness of the wire and decreases from 0.12 eV at the thick edge to 0.09 eV at the thin edge, can be found in Supplementary Fig. 7.

### Size-dependent absorption and emission onset

**Fig. 3:**
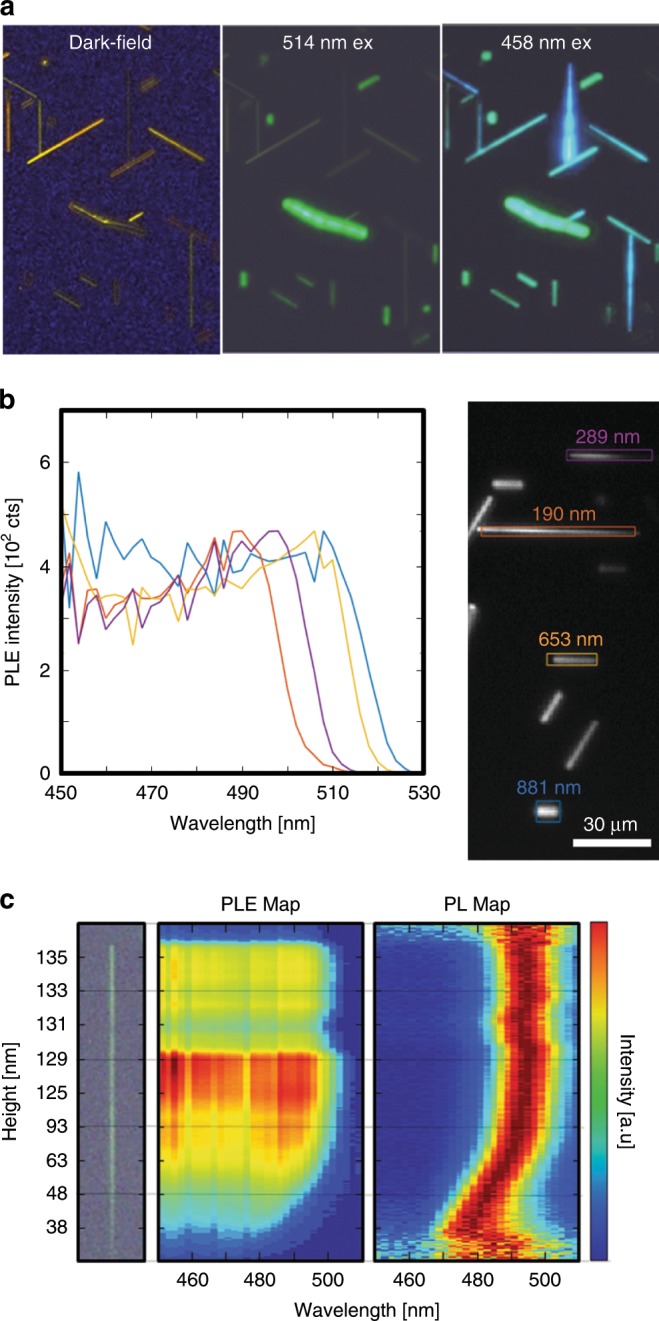
Size-dependent PL-excitation spectra and excitation onset of CsPbBr_3_ nanowires. (**a**) An ensemble of surface-guided CsPbBr_3_ nanowires with different heights, with images showing a dark-field image and PL images with 514 and 458 nm excitation. (**b**) PLE spectra of structures with different heights. The colors of the lines correlate with the heights indicated by the same colors in the transmission image to the right of the plot. A blue-shift in emission excitation onset (sudden rise in PLE intensity upon decreasing the excitation wavelength) with decreasing height can be observed. (**c**) Normalized PL and PLE maps of a single tapered nanowire correlated with its height at each position. Both the peak emission wavelength (PL map) and emission onset wavelength (PLE map) show orchestrated blue-shifts with decreasing height of the nanowire.

In order to evaluate whether the emission shift is the result of a pure optical effect, such as photon reabsorption^4,48^, or an actual bandgap modulation, we examine the optical properties of the nanowires using a combination of spatially resolved excitation and emission spectroscopy. In this excitation configuration, a spectral blue-shift in the emission onset reflects the ability of the nanowires to absorb light of different wavelengths. An ensemble of surface-guided CsPbBr_3_ nanowires with different heights was used in a wide-field excitation experiment. When the nanowire-ensemble is excited with a 514 nm CW laser, only the relatively thick structures emit light. However, when the same ensemble is excited with a 458 nm CW laser, both the thin and the thick structures emit (Fig. 3a). It follows that the spectral onset of the excitation spectra blue-shifts with decreasing height (thickness) of the nanowires in the same way as the emission peak maximum, which effectively signifies that thinner nanowires have higher bandgap energies than thicker nanowires.

To examine the emission onset as a function of the excitation wavelength in detail, we conducted PL-excitation microscopy (PLExMic) experiments on an ensemble of nanowires with various heights. A supercontinuum light source was used to scan the excitation wavelength from 530 to 450 nm, while collecting the emitted light from the low energy tail of the emission (Supplementary Fig. 4 for details). Using this spectroscopic set-up, we measured the band to band emission onset which, according to the semiconductor band theory, corresponds to the absorption onset. Thus, we effectively obtain information on the absorption properties of the nanowires. A height-dependent shift in the emission onset can be observed for an ensemble of nanowires with different heights and for a single nanowire with a height gradient (Fig. 3b, c). We chose four structures with greatly varying heights (Fig. 3b) and collected their PLE spectra. The excitation spectra onset blue-shifts from ~525 nm to ~500 nm with decreasing heights from 881 to 190 nm, which is in full agreement with the trend we observe for the emission peak. We were also able to demonstrate a complete dataset of position-dependent PLE and PL spectra along a single tapered nanowire, shown in Fig. 3c, where the emission onset (PLE) and the emission peak wavelength (PL) can be correlated with the height of the nanowire at each point along its growth axis. The complementary aspects of the PL and PLE measurements and the fact that both datasets reflect the same height to wavelength trend, provide strong experimental evidence that we observe a height-dependent bandgap modulation.

### Direct evidence for unusual lattice distortions

As discussed earlier, the size-dependent emission shift was reported several times in MHPs systems. Prior to our report, a size-dependent emission shift was observed both in MAPbI_3_ platelets and polycrystalline thin films^35,36,49^. The emission shift in the platelets system was rationalized in terms of a surface-induced confinement effect which generates effective potential wells that are smaller than the actual size of the nanostructure^36^. For polycrystalline thin films, the shift was attributed to substrate-induced strain or Pb–I bond stress. Recently, a similar anomalous emission blue-shift was also observed in platelets of CsPbBr_3_ that were grown on a mica substrate^37^. In the latter work, supported by density functional theory (DFT) calculations, the authors proposed that accumulated strain due to nontrivial strength of the van der Waals epitaxial interaction causes the emission shift. Most of these reports point to structural effects as the source for the emission shift. However, they do not provide direct experimental evidence of the lattice structure. Visualization of the lattice with atomic resolution can provide such correlation between the atomic structure and the bandgap modulation that we observe.

Commonly employed explanations for an optical bandgap modulation, such as quantum confinement, surface-induced quantum confinement, thermal expansion and the Burstein–Moss (BM) effect, were all considered but found not compatible with our observations^8^. Here, we also provide spectroscopic evidence that photon reabsorption is not the dominant contributor to the spectral emission blue-shift. Since structural phenomena such as lattice strain and distortions or variance in phase or composition were previously reported both experimentally and theoretically to modulate the bandgap in MHPs^50–54^, we set out to scrutinize the crystal structure and composition of the surface-guided CsPbBr_3_ nanowires in order to determine their role in the observed bandgap modulation. For this purpose, we employed high-resolution STEM techniques to inspect, at the atomic scale, structural or compositional variance in our nanowires that might give rise to the observed bandgap modulation.

**Fig. 4:**
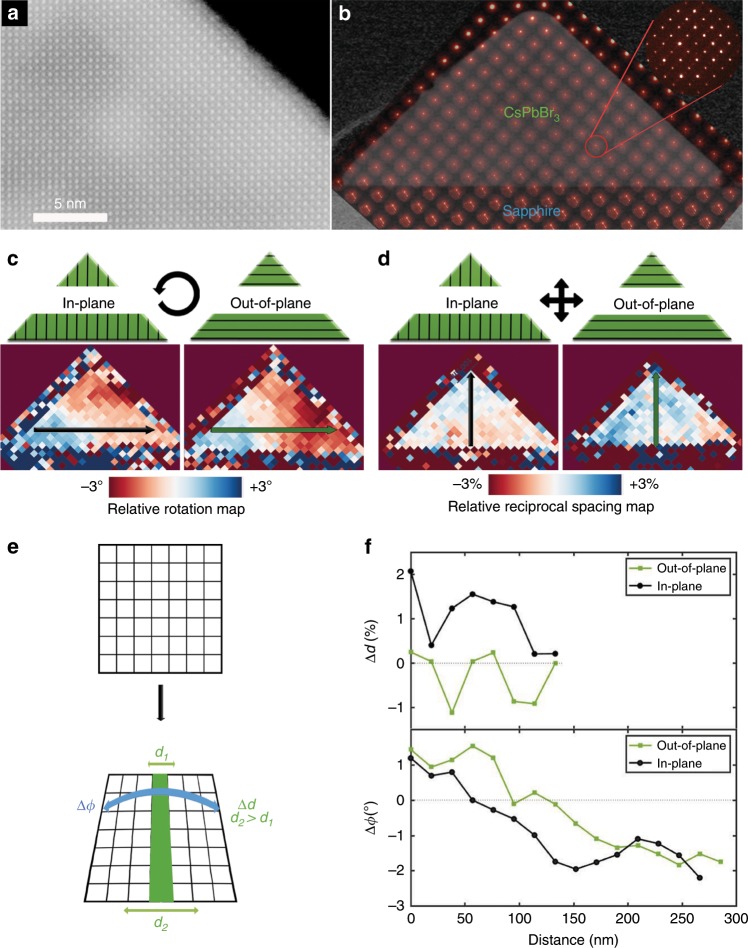
Scanning electron diffraction analysis of surface-guided CsPbBr_3_ nanowires cross-section. (**a**) High magnification image displaying the single-crystal CsPbBr_3_ lattice. (**b**) Nanobeam scanning electron diffraction (SED, or 4D-STEM) analysis, a TEM image overlaid with the actual diffraction patterns acquired at every position across the nanowire cross-section. One typical diffraction pattern is enlarged at the inset. For each nanowire cross-section: (**c**) the in-plane and out-of-plane relative reciprocal-lattice rotation map and (**d**) relative reciprocal-lattice spacing map. (**e**) A schematic model that illustrates the real-space distortion of a cubic lattice according to the trends found in the SED analysis for the in-plane direction: Lattice rotation (Δ*ϕ*) and dilated spacings near the substrate (Δ*d*). (**f**) Line profiles along the black and green arrows indicated in (**c**) of the real-space lattice spacings difference (Δ*d*) and relative lattice rotation (Δ*ϕ*). Source data are provided as a Source Data file.

In order to conduct TEM imaging and analysis, we prepared FIB cross-sectional lamellae of four nanowires with heights between 35 and 220 nm. As discussed earlier, EDS mapping (Fig. 1e and Supplementary Fig. 3) and analysis reveal the expected stoichiometric ratio and a uniform distribution of elements. Our findings, therefore, do not support a compositional variance as the root for the bandgap modulation, at least within the 3% detection limit of our instrument. STEM images at high magnification show some beam damage that can be clearly seen as periodic lighter-contrast spots. However, the lattice of the high quality single-crystal remains generally intact as can be seen at high magnification where the cubic lattice is clearly resolved (Fig. 4a). In fact, scanning over the entire cross section we could not find any visible extended defects.

To examine lattice variations within the cross-section, we employed nanobeam scanning electron diffraction (SED, or 4D-STEM). An electron beam with a 3–10 nm diameter was scanned in a rectangular raster frame over the entire nanowire while collecting a two-dimensional diffraction pattern, in transmission, at each raster position (Fig. 4b and Supplementary Figs. 8, 9). This 4D-STEM diffraction dataset was analyzed for the reciprocal-lattice spacings and rotation angles of in-plane and out-of-plane CsPbBr_3_ lattice planes. Fig. 4c, d and Supplementary Fig. 8 show maps of the reciprocal rotation angle relative to the substrate and lattice spacings relative to the corresponding bulk values, parallel (out-of-plane) and normal (in-plane) to the substrate across the whole nanowire. Qualitatively, we see similar trends in the lattice distortion of thick and thin nanowires. The reciprocal rotation maps reveal an in-plane and out-of-plane lattice rotation (Δ*ϕ*) in all the nanowire cross-sections that were analyzed (Fig. 4c and Supplementary Fig. 9). The reciprocal-lattice spacing maps show no distinct trend in the out-of-plane lattice spacing, while the in-plane maps reveal that the reciprocal-lattice spacing is smaller at the interface and becomes larger towards the apex of the triangular cross-section (Fig. 4d). This translates to larger real-space lattice spacing close to the interface (Δ*d*), which points to heteroepitaxial-induced tensile strain. While there is no apparent trend in the out-of-plane lattice spacings from the interface to the apex, the in-plain lattice spacings exhibit decreasing values. This lattice expansion tends to penetrate further into the cross section for large nanowires compared to the small nanowires (Supplementary Fig. 9). Overall, the trends observed in the in-plane direction can be reflected in a schematic model that combines the real-space lattice rotation and the dilated spacings near the substrate that contract towards the apex (Fig. 4e).

In order to address these trends in a more quantitative manner, we have also calculated line profiles of the in-plane and out of plane real-space lattice parameters difference (Δ*d*) and relative lattice rotation angles (Δ*ϕ*). The line profiles were taken along the directions indicated by black and green arrows for the nanowire presented in Fig. 4c, and are plotted in Fig. 4f. The in-plane lattice spacings are dilated near the interface (~4.20 Å) and decrease in a linear manner to ~4.15 Å close to the apex, where the lattice parameter is similar to that of the bulk (110) plane of cubic CsPbBr_3_^55^. More strikingly, in both the in-plane and out-of-plane direction, the lattice rotates more than 1° every 100 nm in a linear manner. In order to evaluate the lattice distortions for nanowires with different heights, we extract line profiles for all four nanowires with heights between 34 and 219 nm (Supplementary Fig. 10). We define the lattice parameter gradient as the change in the lattice parameter (Δ*d*) for a unit length, and similarly the lattice rotation gradient as the change in rotation angle for a unit length. These gradients appear to grow larger as the height of the nanowires decrease and remarkably strong lattice rotations are observed for thinner nanowires. In one case, as much as a 3.5° rotation is recorded across ~80 nm. For further Supplementary discussion and data, see Supplementary Figs 9–10.

**Fig. 5:**
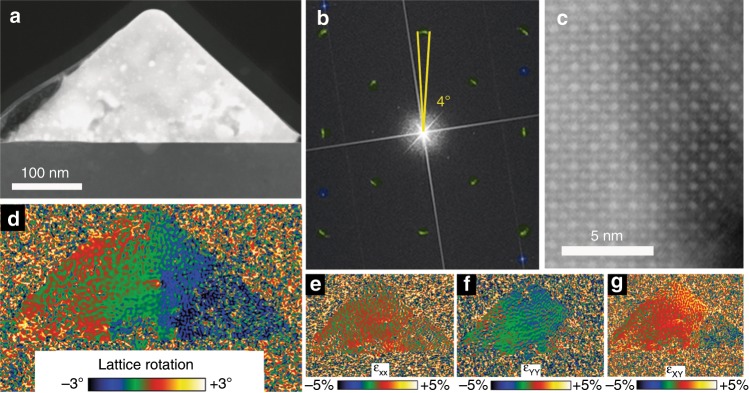
Atomic scale imaging and geometric phase analysis (GPA) of surface-guided CsPbBr_3_ nanowires. (**a**) Large field-of-view, high-resolution STEM image of the entire cross-section. (**b**) Fast Fourier transform of the entire cross-section revealing arcs instead of spots for CsPbBr_3_ spacings (green), and sharp single spots for sapphire spacings (blue). (**c**) High magnification image of the CsPbBr_3_ lattice. (**d**) GPA generated lattice rotation map and maps for in-plane (**e**), out-of-plane (**f**), and shared (**g**) strain.

After observing this unusual lattice distortion with a 5–10 nm resolution limit, we used state-of-the-art aberration-corrected STEM to resolve the strain at the atomic level as displayed in Fig. 5. A large-area and high-resolution STEM image (Fig. 5a) was taken in order to analyze the lattice strain of the whole cross-section using geometrical phase analysis (GPA). Multiple images at higher magnification were also taken at various locations of the cross-section (Supplementary Fig. 11). A quasi-continuous rotation of the lattice can be observed in the fast Fourier transform (FFT) of the whole cross section image (Fig. 5b) as arcs are observed instead of single peaks for lattice spacings that correspond to the perovskite crystal (green), whereas for spacings that match to the sapphire substrate, we observe the expected single sharp peaks (blue). The arc indicates a ~4° rotation of the lattice, which is slightly higher than the value measured with the SED measurements. These differences probably emerge due to the inherent resolution differences between the two methods. Through GPA, we generate the lattice rotation map (Fig. 5d), which highlights a continuous lattice rotation from left to right. This lattice behavior resembles plane bending, which is an elastic stress relief mechanism that is known to emerge in some systems due to heteroepitaxial-induced strain^56^. However, plane bending is usually much more localized at lattice defects and does not spread homogenously across the entire cross-section as reflected in the lattice rotation and strain maps (Fig. 5e, f)^27,56^. A more comparable lattice behavior was recently reported by Tang et al. in a multilayer oxide perovskite BiFeO_3_\LaALO_3_ nanostructure^57^. They observed giant lattice rotations of ~4° across 70 nm that originate from arrays of dislocations at the interface with the substrate in accordance with a partial disclination concept. We could not atomically resolve the sapphire and CsPbBr_3_ interface (Supplementary Fig. 11) and therefore could not observe interfacial defects such as misfit dislocations. However, the remarkably similar lattice behavior and almost identical magnitude of the lattice rotation observed by Tang et al. could imply that an array of misfit dislocation does exist at the CsPbBr_3_–sapphire interface and induces the continuous lattice rotation we observe.

The existence of this unusual elastic stress relief mechanism fits well with the softness and the dynamic nature of MHPs^58,59^. However, during our analysis of the TEM/STEM images we have also occasionally observed FFT peaks that correspond to lattice distances that match half a unit cell (Supplementary Fig. 10) for which we could not unambiguously determine the origin. Therefore, other structural models should also be considered, including one that suggests the coexistence of subdomains and dynamic rotations of the octahedra within nanostructures that seem single crystalline^59^. The origin of theses added peaks is most likely anti-phase domain boundaries, Ruddlesden−Popper phases or nanotwins^59–61^, however considering that the lattice distortion invokes symmetry breaking, which could also play a role in this observation, further research is needed to determine the nature of this observation.

## Discussion

The CsPbBr_3_ surface-guided nanowires have epitaxial relations with the sapphire substrate as described earlier and presented in Fig. 1b, c. Using bulk lattice parameters as reference, the calculated mismatch in the in-plane direction between the (110) plane of CsPbBr_3_ and the $\left( {1\bar 100} \right)$ plane of sapphire at room temperature is +0.7%. This heteroepitaxial mismatch can result in lattice strain and implies that the CsPbBr_3_ lattice is dilated along the in-plane direction at the interface with sapphire^37,39,49^. Since the CsPbBr_3_ nanowires grow on sapphire at ~360 °C before being rapidly cooled down to room temperature, we should also take into account the difference in their expansion coefficients, which could also contribute to the effective mismatch in this system. This effect could be stronger for soft semiconductors like halide perovskites compared to classical semiconductors. The thermal expansion coefficient of CsPbBr_3_ (1.2 × 10^−4^ K^−1^)^50^ is ca. 15 times larger than that of sapphire (8.1 × 10^−6^ K^−1^)^62^. For comparison, the thermal expansion coefficient of GaAs is 6.86 × 10^−6^ K^−1 ^^63^, which is 21 times smaller than that of CsPbBr_3_, and quite similar to that of sapphire. At the synthesis temperature (360 °C) both lattices are expanded and anchored to each other. When cooled down rapidly, both lattices tend to contract to their characteristic room temperature spacings, however the substantially smaller expansion coefficient of sapphire limits the contraction of the CsPbBr_3_ lattice, potentially resulting in a larger (+1.7%) effective mismatch (calculated by applying the reported expansion coefficients of CsPbBr_3_ and sapphire). According to these calculation, the lattice parameter of the in-plane CsPbBr_3_ planes should be dilated near the CsPbBr_3_-sapphire interface inducing tensile strain along the in-plane direction. The observed dilated in-plane lattice spacings of CsPbBr_3_ at the interface with sapphire in our SED analysis (Fig. 4 and Supplementary Fig. 9) are in good agreement with these mismatch calculations and the expected lattice behavior.

**Fig. 6:**
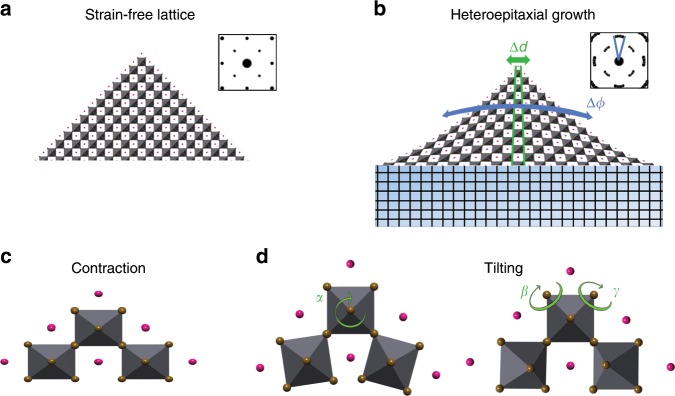
Illustration of the lattice distortion mechanism. (**a**) The CsPbBr_3_ strain free lattice with a diffraction pattern (inset) that indicates their cubic crystal structure. (**b**) Heteroepitaxial nanowire growth causing dilated in-plane lattice spacings (Δ*d*) near the interface resulting in significant lattice rotation (Δ*ϕ*) indicated also by the arcs in the diffraction pattern (inset). The imposed compressive strain in the out-of-plane direction can be accommodated by the CsPbBr_3_ lattice through (**c**) lattice contraction and (**d**) octahedral tilting indicated by *α*, *β*, and *γ*. In all illustrations: Pb atoms are not visible but are located in the center of the gray octahedra, brown spheres represent Br atoms and pink spheres represent Cs atoms.

Figure 6 schematically depicts the strain free CsPbBr_3_ lattice as it grows without a substrate (Fig. 6a) and a distorted lattice due to mismatched heteroepitaxial growth (Fig. 6b). With hard semiconductors, the tensile in-plane strain is often compensated in out-of-plane contraction (Fig. 6c)^64^. Otherwise, the heteroepitaxial stress is relieved via misfit dislocation or elastic plane bending^27,56,65^. Perovskites can accommodate an in-plane tensile or compressive strain also by octahedral tilting or rotation, perpendicular or parallel to the substrate (Fig. 6d)^66–68^.

In order to understand the bandgap increase with decreasing height in cubic CsPbBr_3_ nanowires, we first analyze how the height of the nanowires affects the lattice distortion profile and later assess how the observed profiles should impact the bandgap energy. In our analyses we find that: (i) The in-plane lattice parameter is dilated (Δ*d* ~ 2%) at the interface with sapphire and contracts towards the apex. As the height of the nanowire decreases, the in-plane lattice parameter gradient tends to increase (Supplementary Fig. 10). (ii) Large lattice rotations (Δ*ϕ* ~ 4°) are observed along the in-plane and out-of-plane lattice directions. As the height of the nanowire decreases, larger lattice rotation gradients are observed (Supplementary Fig. 10). Combining these trends, the CsPbBr_3_ lattice seems to be subjected to larger lattice rotation and lattice parameter gradients as the height of the nanowire decreases.

When turning to theory and experimental data from the literature in order to assess how these lattice distortions should affect the bandgap energies of the CsPbBr_3_ nanowires, a complex picture of competing effects arises^54,67,68^. While pressure-induced octahedral tilting, usually decreases the Pb–*X*–Pb (*X*=I, Cl, Br) bond angle, and results in increased bandgaps, pressure-induced contraction of the Pb–*X*–Pb bond generates the opposite effect^67^. In light of the increased bandgap energies we observe in thinner nanowires that are subjected to larger lattice distortion gradients, it is reasonable to qualitatively attribute these bandgap blue-shifts mainly to the large lattice rotations that correlate with strong octahedral tilting and a decrease in the Pb–Br–Pb bond angle.

Owing to the strong contribution of the Pb–Br–Pb bond angle to pressure-induced bandgap shifts, we do not compare our cubic CsPbBr_3_ system with related work done on orthorhombic CsPbBr_3_^54^. Alternatively, for a cubic phase analog, we turn to CsPbI_3_, and specifically to the bandgap engineering of cubic CsPbI_3_ nanocubes under pressure, for further insight^68^. Zou et al. found that cubic CsPbI_3_ exhibit small bandgap red-shifts (from 689 to 691 nm) under low pressure (<0.38 GPa) which induces a ~0.7% contraction of the lattice parameters. Beyond that pressure, and up to 3.5 GPa, the bandgap blue-shifts more rapidly with applied pressure (691–664 nm), governed by lattice distortions that decrease the initial 180° angle of the Pb–I–Pb bond. Correlating these results with our findings, the calculated heteroepitaxial mismatch (0.7–1.7%) in surface-guided CsPbBr_3_ nanowires on sapphire, could induce compressive out-of-plane strain that is comparable to CsPbI_3_ in the high external pressure regime, where the bandgap energies are dominated by the change in the Pb–I–Pb bond angle. The large bandgap blue-shifts (525–490 nm, i.e. up to 35 nm or 0.17 eV) observed in the present work, fit nicely with the expected bandgap behavior in a regime where the bandgap energy is governed by the Pb–Br–Pb bond angle.

Additional experimental data that can contribute to our understanding of the interplay between the lattice distortions and the resulting bandgap shifts are the linewidths of the CL and PL spectra. The FWHM of emission spectra is expected to increase as the system becomes more disordered and less uniform. In both the PL and CL we observe almost no FWHM broadening as the height of the nanowire increases (Supplementary Figs. 5 and 7). This implies that the main structural distortions that are governing the bandgap blue-shifts are distributed fairly uniformly across the lattice. According to the lattice distortion profiles (Figs. 4–5, Supplementary Fig. 10), the relative rotation angle changes in a linear and continuous manner, and could thus be regarded, in a first approximation, as a uniform distortion across the nanowires. The lattice in-plane parameter profiles, in contrast, expose lattice non-uniformity, with larger lattice parameters at the interface with the substrate. This distortion would induce linewidth broadening in the emission spectra unless the charge carriers are always funneled to a region with the lowest bandgap. The only minor narrowing of the emission linewidth as the bandgap blue shifts, implies that the lattice parameter dilatation is not a major contributor to the bandgap modulation in this system.

The atomic-scale visualization of lattice distortions in these epitaxial CsPbBr_3_, along with the lack of variation in structural composition and crystal phase, are experimental observations that strongly support the attribution of this size-dependent bandgap modulation to lattice distortions induced by heteroepitaxial mismatch and lattice relaxation. Strain-induced bandgap modulation is a known phenomenon that is extensively exploited and researched in the semiconductor community^69^. However, the extent of the lattice rotation due to heteroepitaxial strain and lattice relaxation along with the induced bandgap modulation are much larger compared with more conventional semiconductors. This is a direct consequence of the soft character of the halide perovskite system, which can more easily tolerate distortions than hard semiconductors. In addition, the surface-guided nanowires, which are grown heteroepitaxially in a relatively high temperature environment, might be substantially more strained than other MHP systems, as we see in the case of non-epitaxial horizontal nanowires on an amorphous SiO_2_ substrate (Supplementary Fig. 6). The induced lattice distortions and the magnitude of the emission shifts can be considerably higher in our system compared with common thin films. Nevertheless, with further complementary theoretical work, the emission shift could be used as a relatively simple, non-destructive and fast technique to assess strain in other systems including MHPs thin films. Bearing in mind that lattice expansion was recently correlated with high efficiency of perovskite based solar cells^41^, and that strain heterogeneity in polycrystalline thin films was reported to influence the optoelectronic properties and stability of the film^35,39,40,42–45,48^, it seems that strain engineering is crucial for pushing the limits of MHPs-based solar cells and other applications. In that case, a simple and straightforward metric such as the PL signature to probe lattice strain can prove as a powerful tool to evaluate the quality and stability of MHPs thin films and of devices based on them.

In summary, we exploit stable, well-defined epitaxial CsPbBr_3_ nanowires in order to uncover the origin of a fundamental and unusual size-dependent emission behavior. We combine several microscopy and spectroscopy techniques in order to demonstrate the existence of an anomalous height-dependent emission shift using an ensemble of nanowires with different heights and a single tapered nanowire with a height gradient along its growth axis. Using PLE, we show that a bandgap modulation is at the root of this phenomenon while reabsorption cannot account for the observed bandgap shifts. We employ state-of-the-art STEM in order to visualize and reveal the structural origin of the bandgap modulation and find that heteroepitaxial-induced strain and lattice relaxation lead to large and continuous lattice rotation throughout the nanowire’s cross-section. In turn, the induced lattice distortions govern a bandgap modulation that shifts the emission to higher energies with decreasing height. Visualizing strain at the atomic scale and understanding its impact on the optoelectronic properties and stability of MHPs thin films is essential to better understand the behavior and fundamental properties of these materials and to optimize devices based on them.

## Methods

### Material synthesis

The non-catalyzed growth of surface-guided CsPbBr_3_ nanowire was carried out in a three-zone horizontal-tube furnace. The quartz tube reactor was purged with a N_2_ (99.999%, Gordon Gas) and H_2_ (99.99995%, Parker Dominic Hunter H_2_-generator) 7:1 mixture and kept at 300 mbar with a constant 400 sccm flow of the N_2_/H_2_ mixture. The flat sapphire substrates (Roditi int.) did not undergo any preparation process. For the precursor, CsBr and PbBr_2_ powders (both purchased from Sigma-aldrich) were mixed in a 2:1 mole ratio and heated at 390 °C for 20 min in a similar N_2_/H_2_ atmosphere. During the synthesis, the precursor was held at 550 °C in the first heating zone of the furnace, while the samples were placed downstream in the second heating zone and held at 350–390 °C. After a 15 min growth period, the furnace was quickly moved away to allow the rapid cooling down of the source and the sample to room temperature. Further details are described elsewhere^8^.

### Structural characterization

The nanowires were imaged with a high-resolution SEM (Zeiss Sigma 500) equipped with a field emission gun. Images were acquired at 3 kV with an aperture of 30µm using the SE2/InLens detector. AFM (Veeco, Multimode Nanoscope 7.30) is used to collect data regarding the height of the NWs. The images were produced by applying tapping mode in open air, and using 300 or 70 kHz (TESP7, RTESP7) etched Si tips (Nanoprobes). In order to analyze the crystal structure of the nanowires and substrate, a focused-ion beam (FIB, FEI Helios 600 Dual Beam microscope) was used to cut thin (50–100 nm) lamellae across or along the nanowires, which were later inspected under a high-resolution TEM. Nanobeam scanning electron diffraction (SED, or 4D-STEM) data were recorded in a FEI Tecnai F20 Twin TEM (Thermo Fisher Scientific Microscopy Solutions, Hillsboro, USA) under low-dose conditions. SED involves the recording of two-dimensional diffraction patterns for each position in a raster of two-dimensional positions in a scan frame, yielding a four dimensional dataset. For SED, the Tecnai F20 microscope was operated in microprobe mode in conjunction with a small condenser aperture to obtain a beam semi-convergence angle of 0.3 mrad, corresponding to a beam diameter of about 3 nm at an electron energy of 200 keV and at a beam current of 10 pA. The diffraction data were recorded on an Orius 600A CCD camera (Gatan Inc., Pleasanton, USA). Camera exposure and electron probe raster were controlled using a custom-written DigiScan script for Gatan Digital Micrograph. Typical recording times for a raster of 25 × 25 points were about 10 min undersampling of the nanowire cross-sectional area was preferred for the scanning diffraction experiment in order to avoid beam damage causing lattice distortion during exposure of the nanowire to the high-energy electrons. The corresponding dose average for the whole nanowire cross-section was approximately 80 e-/Å^2^, about two orders of magnitude less than for a typical atomic-resolution TEM exposure. Analysis of the 4D dataset in terms of relative reciprocal-lattice spacing and rotation was done using a custom-written script. High-resolution STEM images and EDS maps were recorded in a probe aberration-corrected FEI Titan Themis Z microscope equipped with a Super-X energy dispersive X-ray spectroscopy (EDS) unit. An accelerating voltage of 80 kV was selected to limit the effect of knock on radiation damage. High-resolution STEM images were taken with a semi-convergence angle of 16.7 mrad and a probe current of 20 pA. EDS hyperspectral data were obtained with a with a probe semi- convergence angle of 26.2 mrad a beam current of 250 pA. EDS maps were taken last because of expected radiation damage on the electron entrance surface of the CsPbBr_3_ nanowire. GPA for the quantification of lattice spacings and rotations from high-resolution STEM images was applied through Gatan Digital Micrograph scripting. EDS elemental maps were recorded and processed using the FEI Velox software.

### Optical characterization

To collect SEM-CL spectra we used a Gatan MonoCL Elite system equipped with a retractable diamond-turned mirror. The collected light was imaged in panchromatic or monochromatic mode using a high sensitivity PMT (photomultiplier tube) with a spectral range of 160–930 nm. In addition the collected light could be directed to a monochromator and a CCD for parallel spectroscopy with a spectral range of 200–1100 nm. The CL is installed on a Zeiss GeminiSEM 500, a high-resolution SEM equipped with two modes field emission gun. CL measurements were performed at 3 kV with an aperture of 30µm at analytical gun mode. The simultaneous SEM images were collected using the SE2 detector. PL and PLE measurements were conducted in a home-built microscope based on an inverted Olympus IX71 body (Supplementary Fig. 4)^38^. A supercontinuum light source (SuperK Extreme, NKT Photonics) was used to provide the excitation light for the PLE and the 458 nm line of an Ar^+^ laser for PL measurements. To obtain a wide-field excitation spot, a defocusing lens was introduced to focus the excitation close to the back aperture of the objective (Olympus LucPlanFL 40X, NA = 0.6). Rather than a dichroic mirror, for PLE imaging we employed a broad band pass beamsplitter consisted of a small Al mirror evaporated in the middle of a transparent glass^70^. At the position where the image is formed, we placed a variable slit such that narrow part of the image is passed to the camera (Princeton ProEM CCD). The excitation light reflected off the back surface of the sample is filtered out with a long-pass filter, which we can vary according to the excitation wavelength. Prior to reaching the camera, the image is passed through a transmission grating such that the image that passes through the narrow slit is maintained in the zero order diffraction, while the 1st order diffracted light spreads out laterally on the detector with a distance from the zero order based on photon energy. This effectively yields the spectrum of the light emitted at any point along the slit. Placing the wire parallel to the slit opening and adjusting its width to match the width of the wire, we obtain a spectrum at every point of the wire with a spatial resolution of ~500 nm and a spectral resolution of 2 nm. For PL-excitation (PLE) measurements, we set the long-pass filter to pass only the tail of the emission while scanning the excitation between 450 and 520 nm in 2 nm increments. For PL emission measurements, we used excitation at 458 nm and the long-pass filter set to capture the entire emission peak.

## Supplementary information

Supplementary Information

## Data Availability

All datasets generated during and/or analyzed during the current study are available from the corresponding author on reasonable request. The source data underlying Fig. 4f and Supplementary Figs. 5, 6A–B and 10 are provided as a Source Data file.

## Source data

Source Data

## Code availability

All custom codes used the current study are available from the corresponding author on reasonable request.

## References

[1] Snaith HJ (2013). Perovskites: the emergence of a new era for low-cost, high-efficiency solar cells. J. Phys. Chem. Lett..

[2] Johnston MB, Herz LM (2016). Hybrid perovskites for photovoltaics: charge-carrier recombination, diffusion, and radiative efficiencies. Acc. Chem. Res..

[3] Stranks SD (2013). Electron-hole diffusion lengths exceeding 1 micrometer in an organometal trihalide perovskite absorber. Science.

[4] Pazos-Outón LM (2016). Photon recycling in lead iodide perovskite solar cells. Science.

[5] Deschler F (2014). High photoluminescence efficiency and optically pumped lasing in solution-processed mixed halide perovskite semiconductors. J. Phys. Chem. Lett..

[6] Shi D (2015). Low trap-state density and long carrier diffusion in organolead trihalide perovskite single crystals. Science.

[7] Kang J, Wang L-W (2017). High defect tolerance in lead halide perovskite CsPbBr_3_. J. Phys. Chem. Lett..

[8] Oksenberg E, Sanders E, Popovitz-Biro R, Houben L, Joselevich E (2018). Surface-guided CsPbBr_3_ perovskite nanowires on flat and faceted sapphire with size-dependent photoluminescence and fast photoconductive response. Nano Lett..

[9] Zhu H (2015). Lead halide perovskite nanowire lasers with low lasing thresholds and high quality factors. Nat. Mater..

[10] Tan Z-K (2014). Bright light-emitting diodes based on organometal halide perovskite. Nat. Nanotechnol..

[11] Zhang C (2015). Magnetic field effects in hybrid perovskite devices. Nat. Phys..

[12] Xing G (2014). Low-temperature solution-processed wavelength-tunable perovskites for lasing. Nat. Mater..

[13] Egger DA (2018). What remains unexplained about the properties of halide perovskites?. Adv. Mater..

[14] deQuilettes DW (2016). Photo-induced halide redistribution in organic–inorganic perovskite films. Nat. Commun..

[15] Shao Y (2016). Grain boundary dominated ion migration in polycrystalline organic–inorganic halide perovskite films. Energy Environ. Sci..

[16] Stranks SD (2017). Nonradiative losses in metal halide perovskites. ACS Energy Lett..

[17] Eaton SW (2016). Lasing in robust cesium lead halide perovskite nanowires. Proc. Natl Acad. Sci. U. S. A..

[18] Deng W (2017). Ultrahigh-responsivity photodetectors from perovskite nanowire arrays for sequentially tunable spectral measurement. Nano Lett..

[19] Yuan C (2018). Chiral lead halide perovskite nanowires for second-order nonlinear optics. Nano Lett..

[20] Shoaib M (2017). Directional growth of ultralong CsPbBr_3_ perovskite nanowires for high-performance photodetectors. J. Am. Chem. Soc..

[21] Park K (2016). Light–matter interactions in cesium lead halide perovskite nanowire lasers. J. Phys. Chem. Lett..

[22] Wang Y (2016). Photon transport in one-dimensional incommensurately epitaxial CsPbX_3_ arrays. Nano Lett..

[23] Chen J (2017). Vapor-phase epitaxial growth of aligned nanowire networks of cesium lead halide perovskites (CsPbX_3_, X = Cl, Br, I). Nano Lett..

[24] Zhou H (2017). Vapor growth and tunable lasing of band gap engineered cesium lead halide perovskite micro/nanorods with triangular cross section. ACS Nano.

[25] Xu, J., Oksenberg, E., Popovitz-Biro, R., Rechav, K. & Joselevich, E. Bottom-up tri-gate transistors and submicrosecond photodetectors from guided CdS nanowalls. *J. Am. Chem. Soc*. **139**, 15958–15967 (2017). 10.1021/jacs.7b09423 29035565

[26] Shalev E, Oksenberg E, Rechav K, Popovitz-Biro R, Joselevich E (2017). Guided CdSe nanowires parallelly integrated into fast visible-range photodetectors. ACS Nano.

[27] Oksenberg E, Martí-Sánchez S, Popovitz-Biro R, Arbiol J, Joselevich E (2017). Surface-guided core–shell ZnSe@ZnTe nanowires as radial p–n heterojunctions with photovoltaic behavior. ACS Nano.

[28] Neeman L (2017). Crystallographic mapping of guided nanowires by second harmonic generation polarimetry. Nano Lett..

[29] Oksenberg E, Popovitz-Biro R, Rechav K, Joselevich E (2015). Guided growth of horizontal ZnSe nanowires and their integration into high-performance blue-UV photodetectors. Adv. Mater..

[30] Tsivion D, Schvartzman M, Popovitz-Biro R, von Huth P, Joselevich E (2011). Guided growth of millimeter-long horizontal nanowires with controlled orientations. Science.

[31] Miao X (2015). High-speed planar GaAs nanowire arrays with fmax > 75 GHz by wafer-scale bottom-up growth. Nano Lett..

[32] Choi W (2017). Direct electrical probing of periodic modulation of zinc-dopant distributions in planar gallium arsenide nanowires. ACS Nano.

[33] Leppert L, Reyes-Lillo SE, Neaton JB (2016). Electric field- and strain-induced Rashba effect in hybrid halide perovskites. J. Phys. Chem. Lett..

[34] Kulbak M (2016). Cesium enhances long-term stability of lead bromide perovskite-based solar cells. J. Phys. Chem. Lett..

[35] D’Innocenzo V, Srimath Kandada AR, De Bastiani M, Gandini M, Petrozza A (2014). Tuning the light emission properties by band gap engineering in hybrid lead halide perovskite. J. Am. Chem. Soc..

[36] Li D (2016). Size-dependent phase transition in methylammonium lead iodide perovskite microplate crystals. Nat. Commun..

[37] Wang Y (2018). Nontrivial strength of van der Waals epitaxial interaction in soft perovskites. Phys. Rev. Mater..

[38] Merdasa A (2019). Impact of Excess Lead Iodide on the Recombination Kinetics in Metal Halide Perovskites. ACS Energy Lett..

[39] Zhao Jingjing, Deng Yehao, Wei Haotong, Zheng Xiaopeng, Yu Zhenhua, Shao Yuchuan, Shield Jeffrey E., Huang Jinsong (2017). Strained hybrid perovskite thin films and their impact on the intrinsic stability of perovskite solar cells. Science Advances.

[40] Phung, N. & Abate, A. the impact of nano- and microstructure on the stability of perovskite solar cells. *Small* **0**, 1802573. 10.1002/smll.201802573 30295009

[41] Tsai H (2018). Light-induced lattice expansion leads to high-efficiency perovskite solar cells. Science.

[42] Pandya S (2016). Strain-induced growth instability and nanoscale surface patterning in perovskite thin films. Sci. Rep..

[43] Zheng X (2016). Improved phase stability of formamidinium lead triiodide perovskite by strain relaxation. ACS Energy Lett..

[44] Saidaminov MI (2018). Suppression of atomic vacancies via incorporation of isovalent small ions to increase the stability of halide perovskite solar cells in ambient air. Nat. Energy.

[45] Jones TW (2019). Lattice strain causes non-radiative losses in halide perovskites. Energy Environ. Sci..

[46] Steele JA (2019). Thermal unequilibrium of strained black CsPbI3 thin films. Science.

[47] Protesescu L (2015). Nanocrystals of cesium lead halide perovskites (CsPbX_3_, X = Cl, Br, and I): novel optoelectronic materials showing bright emission with wide color gamut. Nano Lett..

[48] Diab H (2017). Impact of reabsorption on the emission spectra and recombination dynamics of hybrid perovskite single crystals. J. Phys. Chem. Lett..

[49] Shojaee SA, Harriman TA, Han GS, Lee J-K, Lucca DA (2017). Substrate effects on photoluminescence and low temperature phase transition of methylammonium lead iodide hybrid perovskite thin films. Appl. Phys. Lett..

[50] Stoumpos CC (2013). Crystal growth of the perovskite semiconductor CsPbBr3: a new material for high-energy radiation detection. Cryst. Growth Des..

[51] Osherov A (2016). The impact of phase retention on the structural and optoelectronic properties of metal halide perovskites. Adv. Mater..

[52] Yang RX, Skelton JM, da Silva EL, Frost JM, Walsh A (2017). Spontaneous octahedral tilting in the cubic inorganic cesium halide perovskites CsSnX_3_ and CsPbX_3_ (X = F, Cl, Br, I). J. Phys. Chem. Lett..

[53] Bechtel JS, Van der Ven A (2018). Octahedral tilting instabilities in inorganic halide perovskites. Phys. Rev. Mater..

[54] Xiao G (2017). Pressure effects on structure and optical properties in cesium lead bromide perovskite nanocrystals. J. Am. Chem. Soc..

[55] Rodová M, Brožek J, Knížek K, Nitsch K (2003). Phase transitions in ternary caesium lead bromide. J. Therm. Anal. Calorim..

[56] de la Mata M, Magén C, Caroff P, Arbiol J (2014). Atomic scale strain relaxation in axial semiconductor III–V nanowire heterostructures. Nano Lett..

[57] Tang YL, Zhu YL, Liu Y, Wang YJ, Ma XL (2017). Giant linear strain gradient with extremely low elastic energy in a perovskite nanostructure array. Nat. Commun..

[58] Yaffe O (2017). Local polar fluctuations in lead halide perovskite crystals. Phys. Rev. Lett..

[59] Bertolotti F (2017). Coherent nanotwins and dynamic disorder in cesium lead halide perovskite nanocrystals. ACS Nano.

[60] Zhang D, Eaton SW, Yu Y, Dou L, Yang P (2015). Solution-phase synthesis of cesium lead halide perovskite nanowires. J. Am. Chem. Soc..

[61] Yu, Y., Zhang, D. & Yang, P. Ruddlesden–Popper phase in two-dimensional inorganic halide perovskites: a plausible model and the supporting observations. *Nano Lett*. **17**, 5489–5494 (2017). 10.1021/acs.nanolett.7b02146 28796526

[62] Yim WM, Paff RJ (1974). Thermal expansion of AlN, sapphire, and silicon. J. Appl. Phys..

[63] Pierron ED, Parker DL, McNeely JB (1967). Coefficient of Expansion of GaAs, GaP, and Ga(As, P) Compounds from −62° to 200 °C. J. Appl. Phys..

[64] Trushin O, Jalkanen J, Granato E, Ying SC, Ala-Nissila T (2009). Atomistic studies of strain relaxation in heteroepitaxial systems. J. Phys.: Condens. Matter.

[65] Shi J, Wang X (2010). Strain versus dislocation model for understanding the heteroepitaxial growth of nanowires. J. Phys. Chem. C..

[66] Rondinelli JM, Spaldin NA (2011). Structure and properties of functional oxide thin films: insights from electronic-structure calculations. Adv. Mater..

[67] Jaffe A, Lin Y, Karunadasa HI (2017). Halide perovskites under pressure: accessing new properties through lattice compression. ACS Energy Lett..

[68] Cao Y (2018). Pressure-tailored band gap engineering and structure evolution of cubic cesium lead iodide perovskite nanocrystals. J. Phys. Chem. C..

[69] Smith AM, Nie S (2010). Semiconductor nanocrystals: structure, properties, and band gap engineering. Acc. Chem. Res..

[70] Camacho R, Thomsson D, Yadav D, Scheblykin IG (2012). Quantitative characterization of light-harvesting efficiency in single molecules and nanoparticles by 2D polarization microscopy: Experimental and theoretical challenges. Chem. Phys..

