# Supplementary material for: Large lattice distortions and size-dependent bandgap modulation in epitaxial halide perovskite nanowires
Source: Nat Commun. 2020 Jan 24;11:489. doi: 10.1038/s41467-020-14365-2 (PMC6981217; doi:10.1038/s41467-020-14365-2)
Supplement: Supplementary file 1 — Supplementary Information [file 41467_2020_14365_MOESM1_ESM.pdf]

Supplementary Information

Large Lattice Distortions and Size-Dependent Bandgap Modulation in Epitaxial Halide Perovskite Nanowires

Eitan Oksenberg *et al.*

Top view atomistic model sketch of epitaxial $CsPbBr_3$ nanowire on a sapphire (0001) surface.

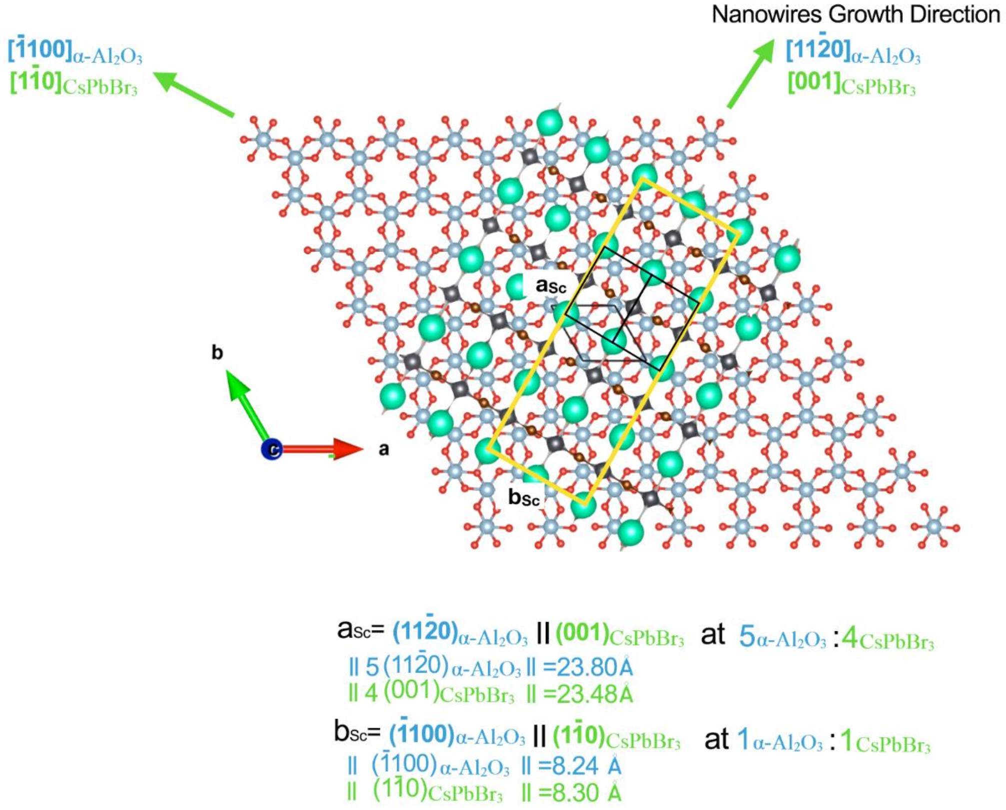

Supplementary Figure 1. Top view of atomistic model showing the interface of sapphire (0001) top surface layer and the $CsPbBr_3$ (110) bottom layer. For sapphire, the Miller-Bravais directions are blue. For $CsPbBr_3$ the Miller directions are in green. The $CsPbBr_3$ grows towards $[11\bar{2}0]$ direction of the sapphire substrate. The interface repeating cell is marked in yellow and the unit cells of sapphire and bromide are sketched in black. a_{Sc} and b_{Sc} represent the interface repeating cell lattice parameters along the $[\bar{1}100]$ viewing direction, and to the orthogonal $[11\bar{2}0]$ direction of the sapphire respectively. Red and gray spheres represent the Al and O atoms, respectively, of the sapphire substrate. Green, dark-gray and brown spheres represent the Cs, Pb and Br atoms of the nanowire.

AFM characterization of a typical nanowire

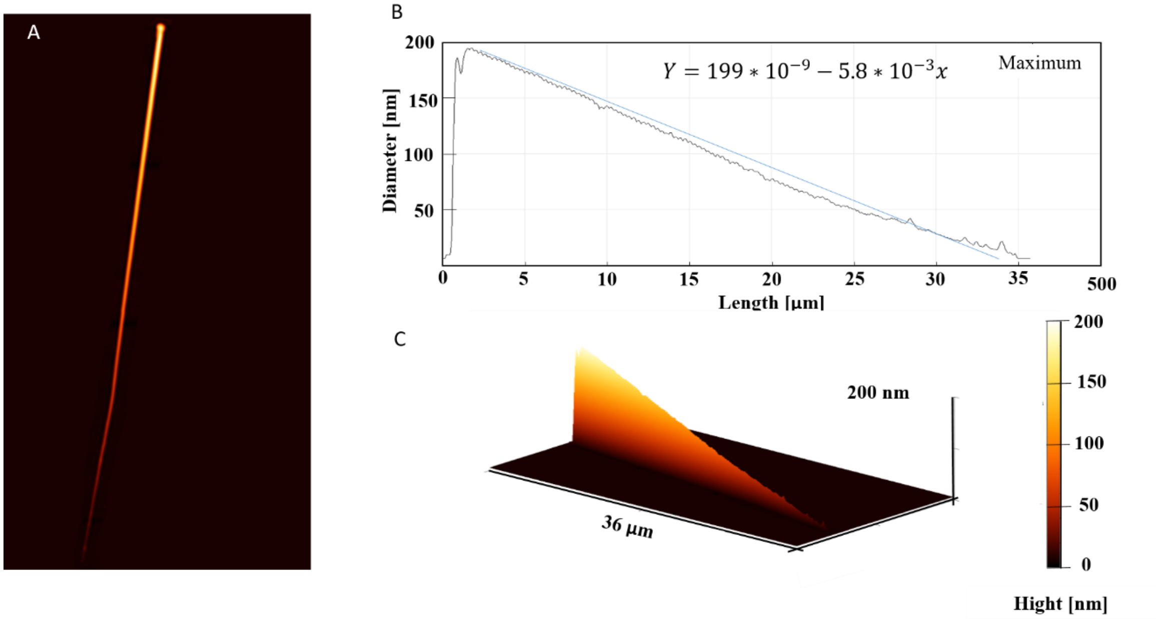

Supplementary Figure 2**.** A typical tapered nanowire with a height gradient displayed in (A) a 2D SFM image. The maximal height value was extracted, plotted (B) and fitted to an almost perfect linear gradient. (C) A 3D AFM images using a color scale-bar depicting the decay from ~200 nm to ~10 nm along a 33 micrometer long nanowire.

EDS elemental mapping and analysis.

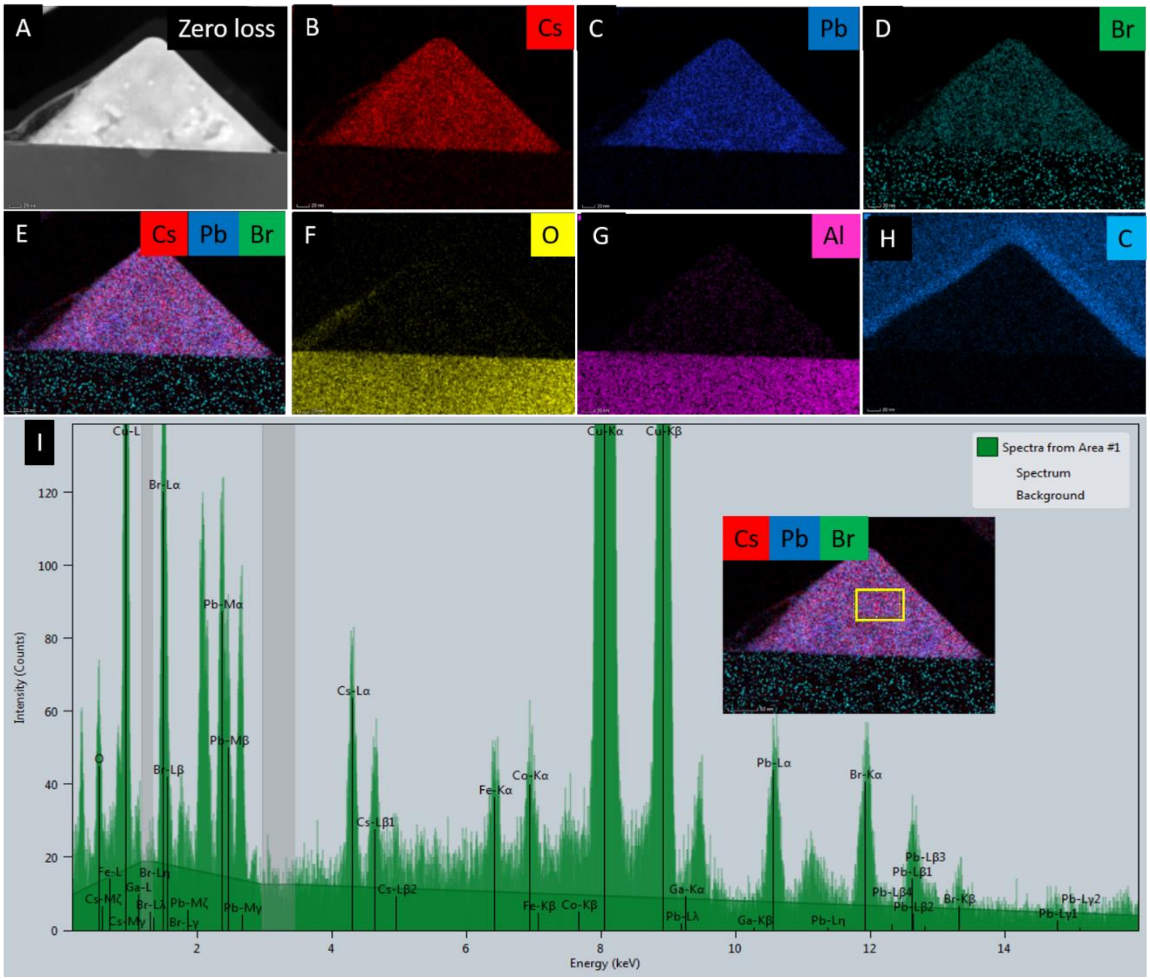

Supplementary Figure 3. EDS elemental mapping and quantification: (A) Zero loss image. (B) Cs map. (C) Pb map. (D) Br map and (E) a combined map of Cs, Pb and Br. Substrate elements, (F) Oxygen map. (G) Al map and a (H) background Carbon map. (I) the EDS spectrum collected from the yellow rectangle within the cross section that is displayed in the inset.

Atomic resolution STEM images and EDS elemental maps were recorded at an electron energy of 80 keV in a double-corrected FEI Themis-Z (S)TEM equipped with a Super-X four-quadrant EDS SDD detector. Geometrical phase analysis for the quantification of lattice spacings and rotations from high-resolution STEM images was applied through Gatan Digital Micrograph scripting. EDS elemental maps were recorded and processed using the FEI Velox software.

PL and PLE set-up and additional data and analysis.

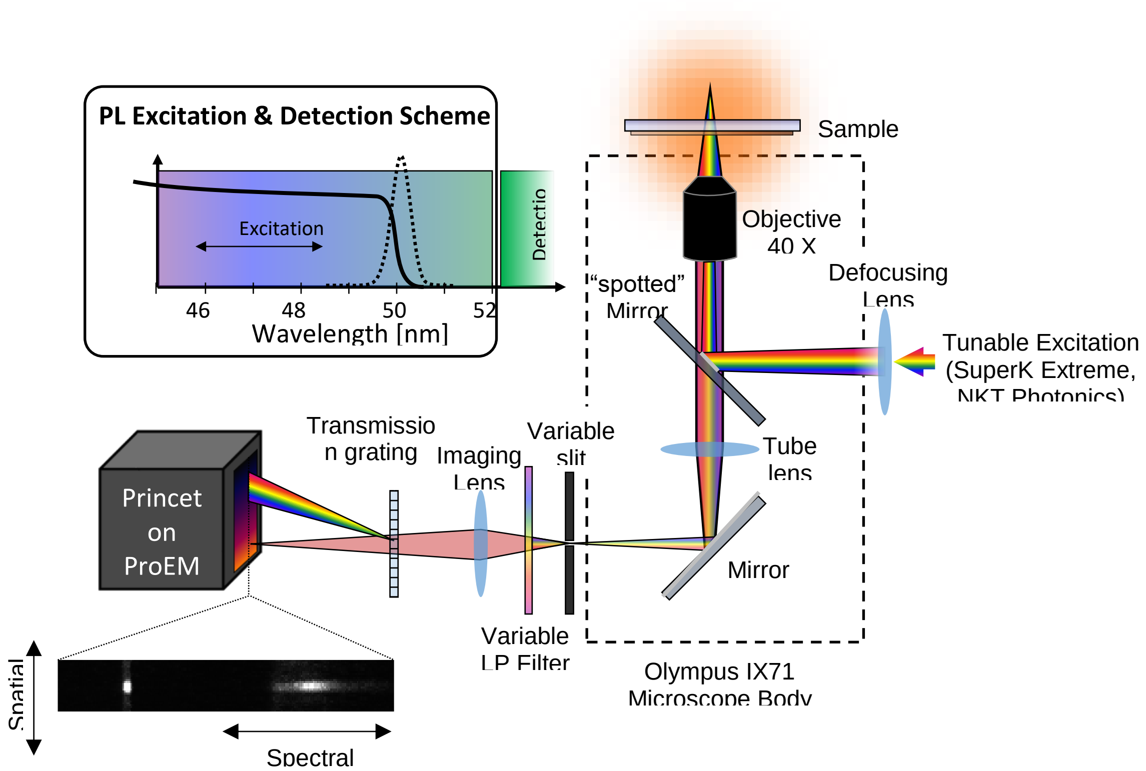

Supplementary Figure 4. Illustration of the optical path and the collected data in PL and PLE measurments.

PL and PLE measurements were conducted in a homebuilt microscope based on an inverted Olympus IX71 body. A supercontinuum light source (SuperK Extreme, NKT photonics) was used to provide the excitation for all measurements. To obtain a wide-field excitation spot, a defocusing lens was introduced to focus the excitation onto the back aperture of the objective (Olympus LucPlanFL 40X, NA=0.6). Rather than a dichroic mirror, we employed a so-called "spotted" mirror where a small region of aluminum is coated onto a larger glass substrate (not drawn to scale). As such, the excitation can be focused onto a small region in the excitation path where the collected emission can largely pass to be detected. This allowed for a broad excitation range to be used. At the position where the image is formed, we placed a variable slit such that narrow part of the image is passed to the camera (Princeton ProEM CCD). The excitation light reflected off the back surface of the sample is filtered out with a long pass filter which we can vary according to the excitation wavelength. Prior to reaching the camera, the image is passed through a transmission grating such that the image that passes through the narrow slit is maintained in the zero order diffraction, while the 1st order diffracted light spreads out laterally on the detector with a distance from the zero order based on photon energy. This effectively yields the spectrum of the light emitted at any point along the slit. Placing the wire parallel to the slit opening and adjusting its width to match the width of the wire, we obtain a spectrum at every point of the wire with a spatial resolution of ~500 nm and a spectral resolution of 2.1 nm.

For PL excitation (PLE) measurements, we set the long-pass filter to pass only the tail of the emission while scanning the excitation between 450 nm – 520 nm in 2 nm increments. The reason for only passing the tail is to resolve the absorption onset in the PLE spectrum.

For PL emission measurements we used excitation at 458 nm and the long-pass filter set to capture the entire emission peak. Simply changing the filter, we were capable of comparing both PL emission and PL excitation of the same points in the same wire.

Emission trends.

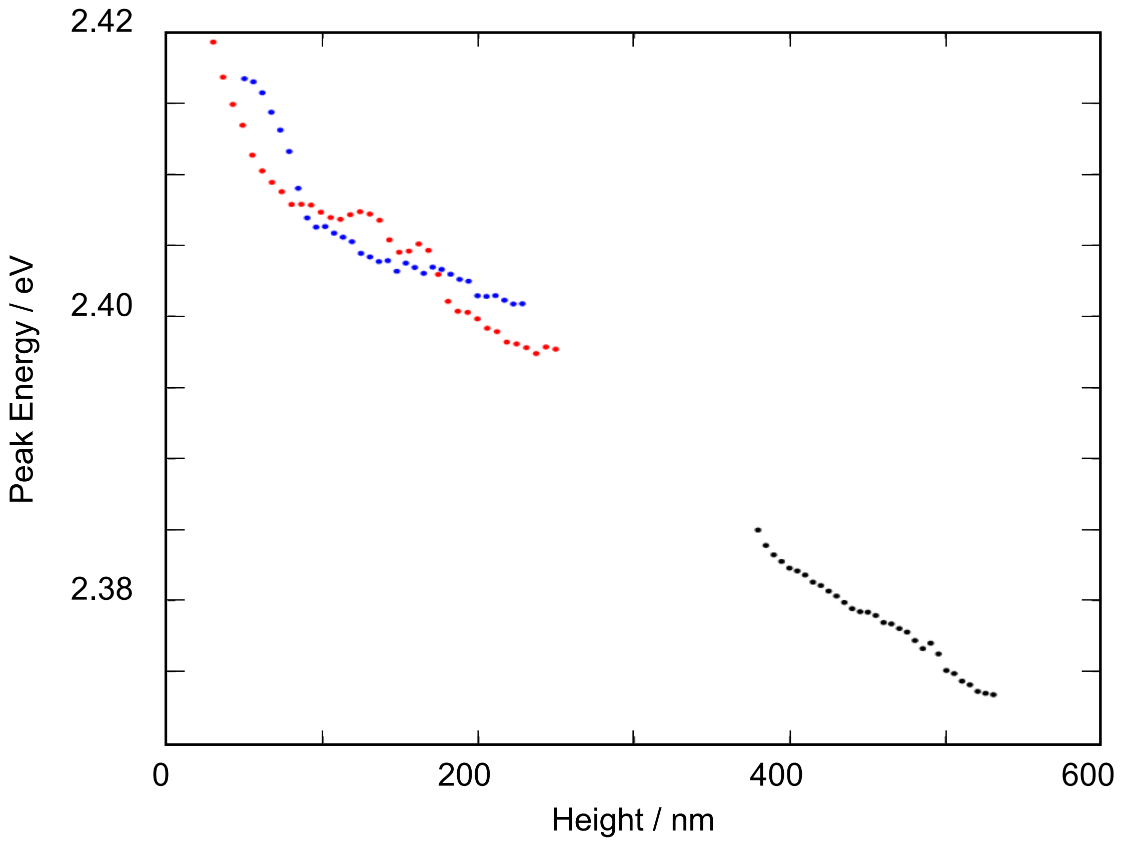

Supplementary Figure 5. Emission trend of tapered nanowires on sapphire. Peak energy of emission versus the height of 3 tapered nanowires grown on sapphire (wire 1-3). The trend has 2 distinctive regimes, a linear regime above ~100 nm and an exponential regime below ~100 nm. Source data are provided as a Source Data file.

Substrate effect on emission trends.

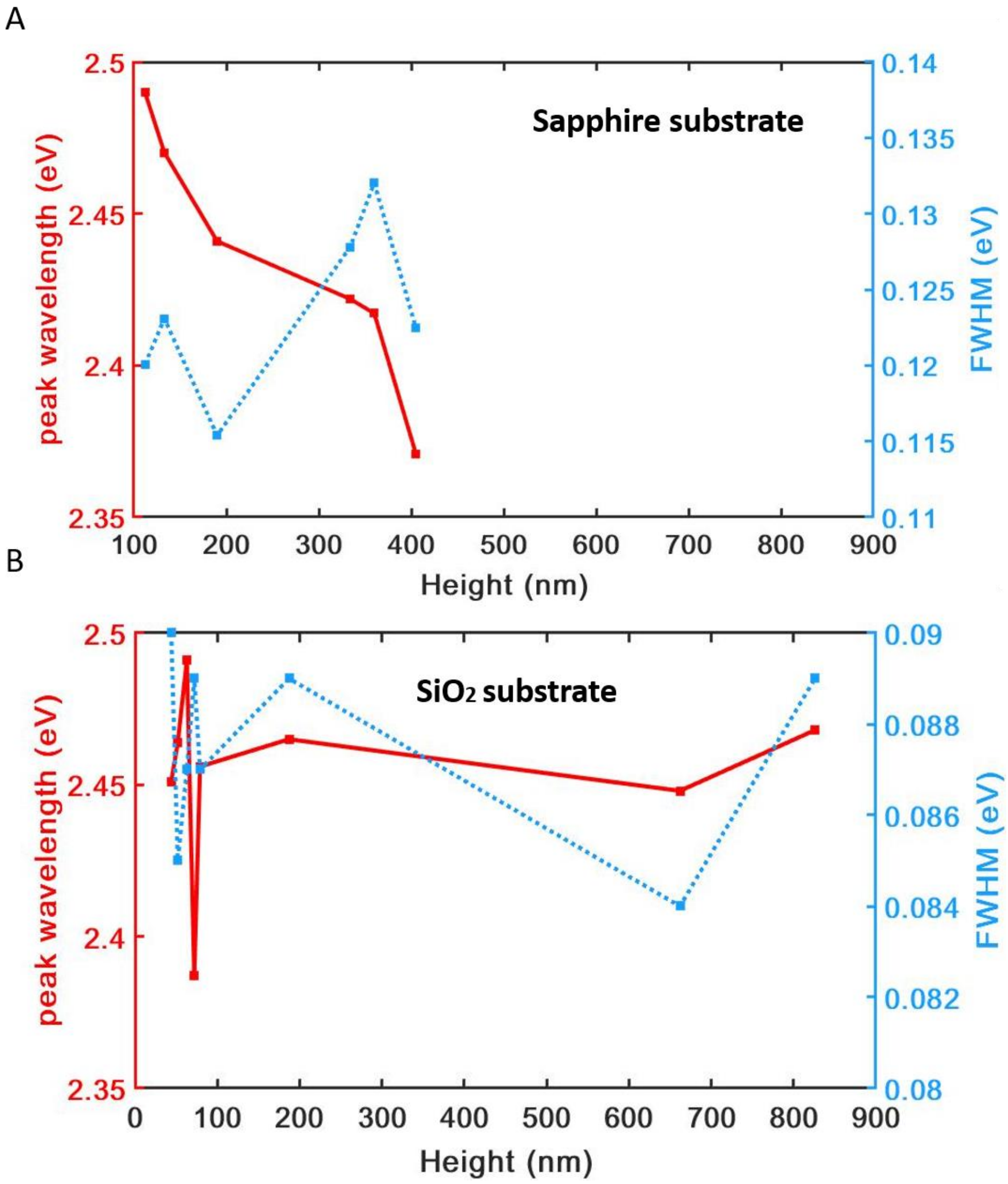

Supplementary Figure 6. Sapphire vs. SiO_2 substrates emission trends. (A) Peak emission wavelength and FWHM versus the height of the 6 nanowires grown epitaxially on sapphire exhibiting a blue-shift of the peak position and FWHM with decreasing heights. The full spectra are presented in figure 2A of the manuscript. (C) Peak emission wavelength and FWHM versus the height of the 9 horizontal nanowires grown on an amorphous SiO_2 substrate exhibiting no apparent height-dependent trend. Source data are provided as a Source Data file.

SEM-Cathodoluminescence of a tapered nanowire.

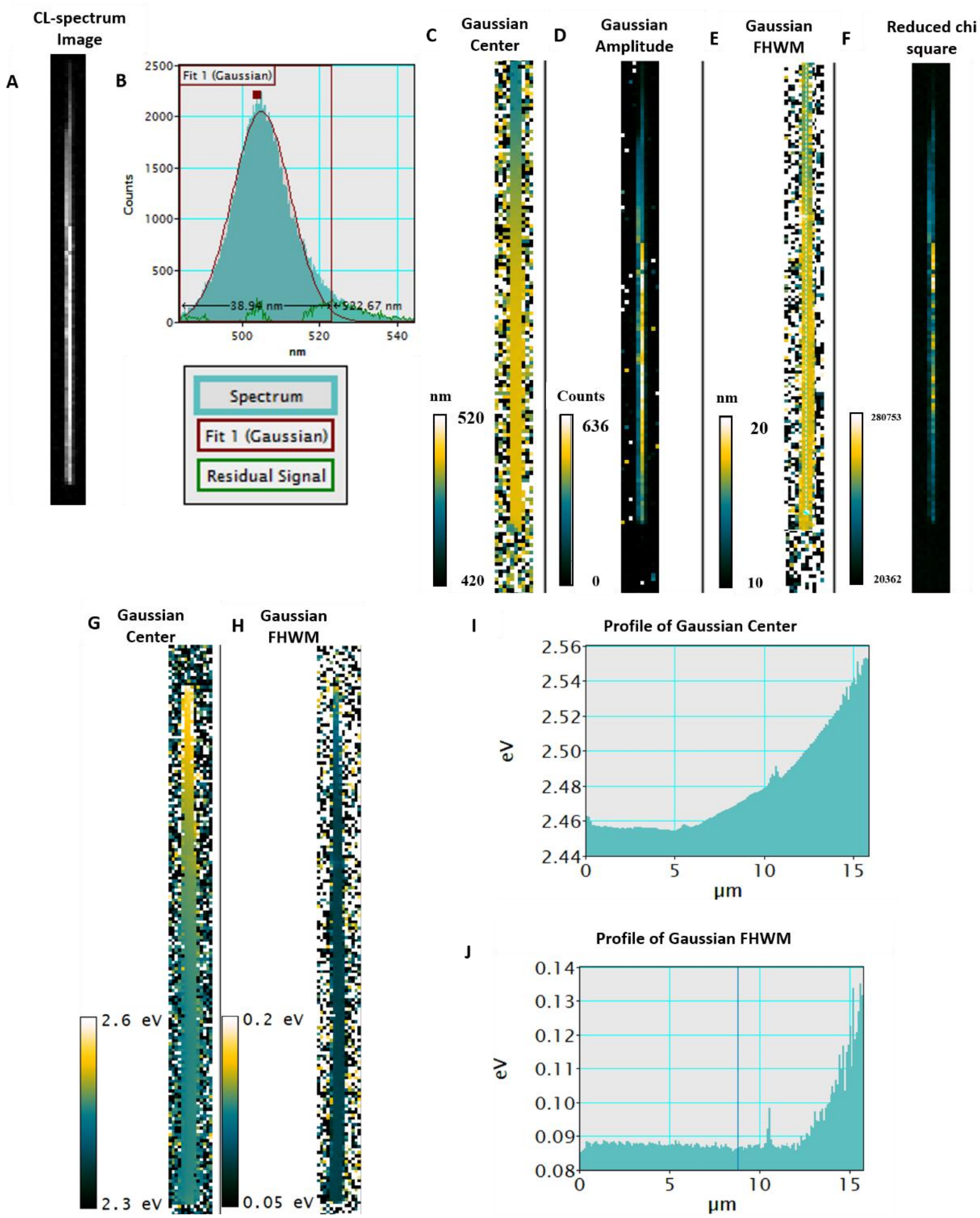

Supplementary Figure 7. CL spectroscopy on a single tapered surface-guided epitaxial $CsPbBr_3$ nanowire (A) CL map: Each 100 nm^2 pixel represents a full CL spectrum (450-520 nm) and the integrated intensity of the emission peak generates the gray-scale contrast of the image. (B) A typical Gaussian fit that is done at each pixel. The extracted wavelength of the emission peak is displayed using a color scale (480-520 nm) to generate the emission peak wavelength map (C). Further data on the Gaussian fit at each pixel: (D) Amplitude map, (E) full width at half-maximum (FWHM) and (F) Reduced chi square. Complementary analysis using energy units: (G) Center and (H) FWHM of the fitted Gaussian (I-J) Line profiles along the axis of the nanowires for the Gaussian center and the FWHM that show the emission blue-shift from the bottom thick edge to the upper thin edge.

Scanning electron diffraction methodology.

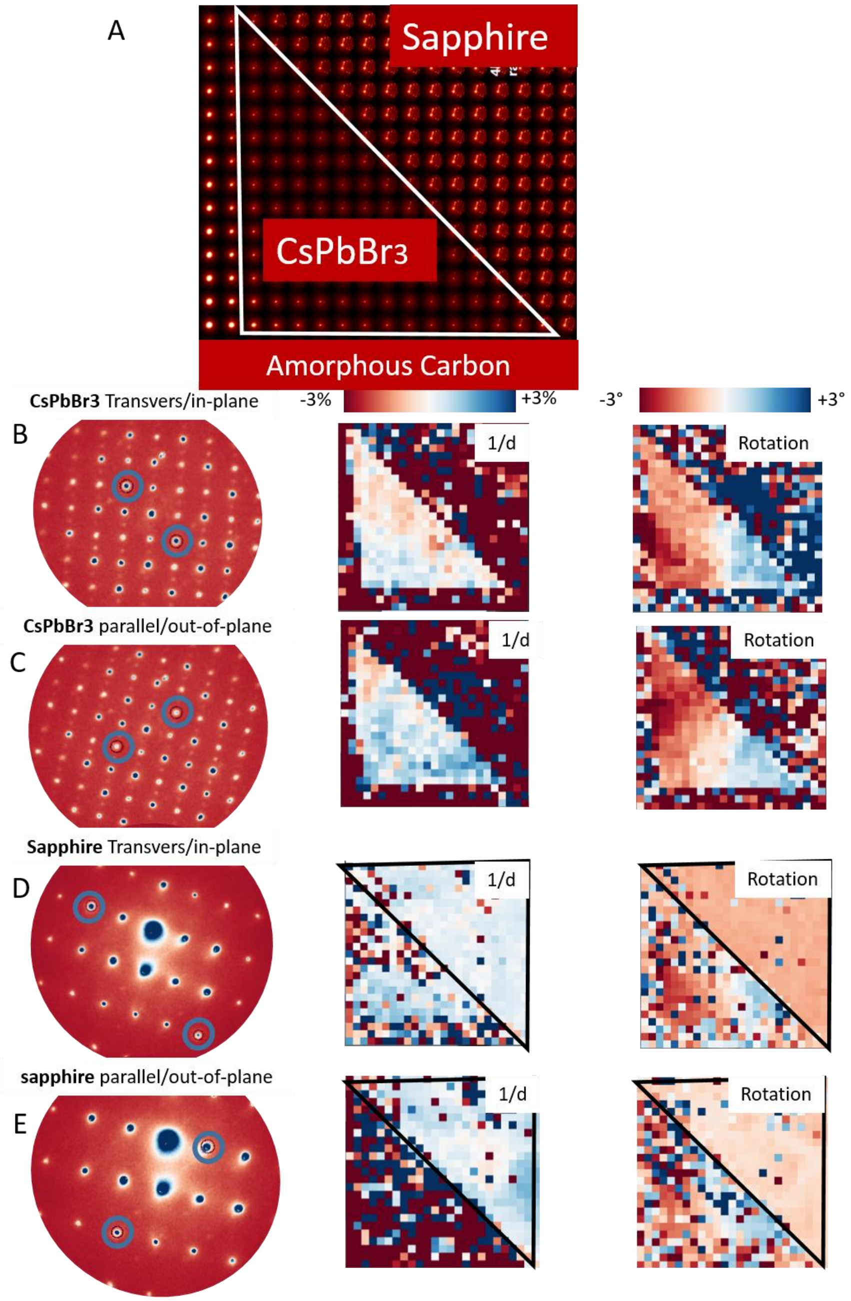

Supplementary Figure 8. Nanobeam scanning electron diffraction (SED, or 4D-STEM) structural analysis of a surface guided $CsPbBr_3$ nanowire. (A) The actual diffractions positioned across the cross-sections with the sapphire, amorphous carbon and $CsPbBr_3$ regions indicated. The selected diffraction spots, and generated (B) in-plane and (C) out-of-plane relative reciprocal-lattice spacing map and the in-plane and out-of-plane relative reciprocal-lattice rotation map for the $CsPbBr_3$ selected planes. The selected diffraction spots, and generated (D) in-plane and (E) out-of-plane relative reciprocal-lattice spacing map and the in-plane and out-of-plane relative reciprocal-lattice rotation map for the sapphire selected planes taken as reference.

Scanning electron diffraction maps for 4 nanowires with heights between 34 nm and 219 nm.

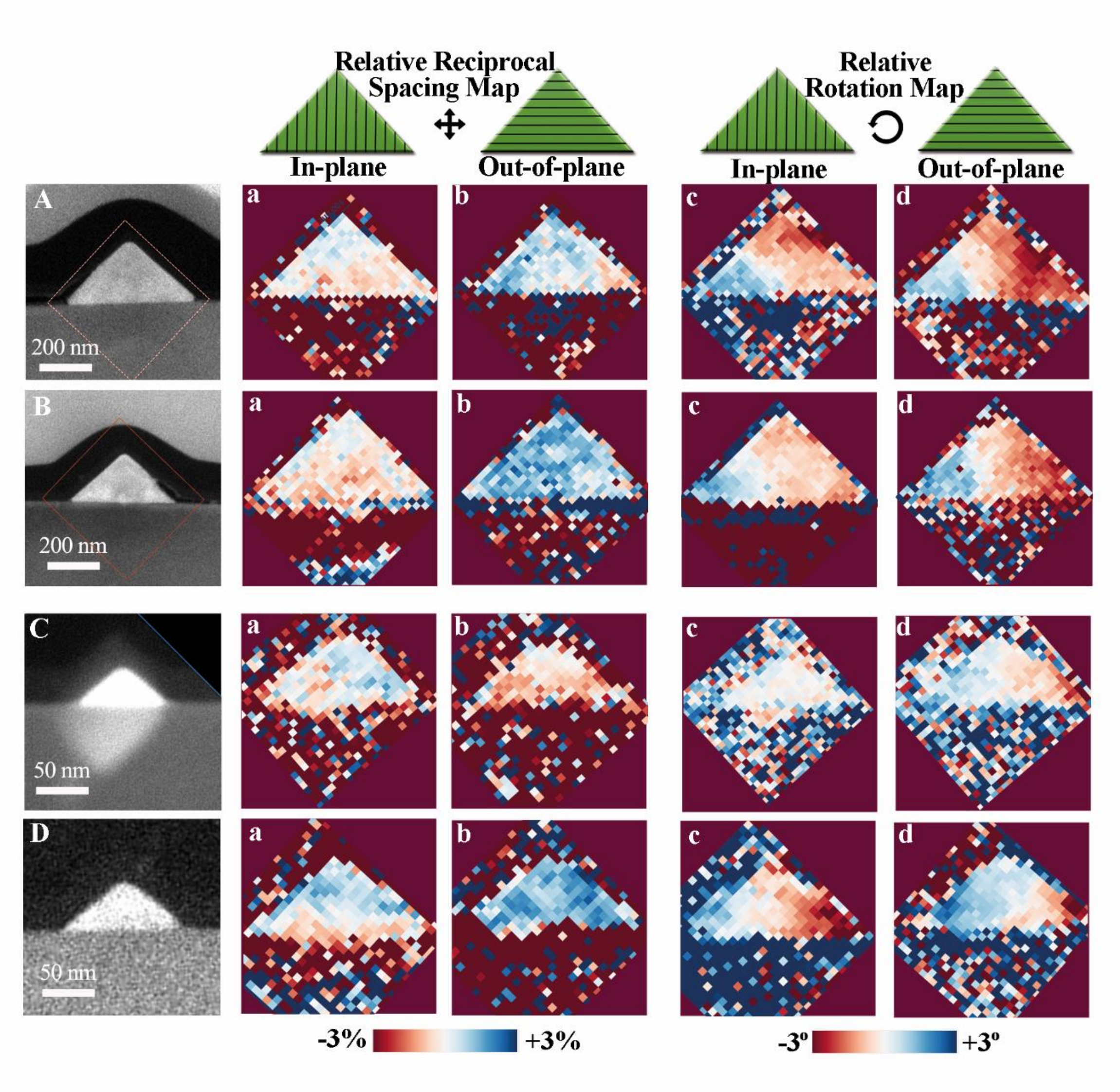

Supplementary Figure 9. Nanobeam scanning electron diffraction (SED, or 4D-STEM) analysis overview of surface guided $CsPbBr_3$ nanowires with large and small heights. For every nanowire cross-section (A-D) the corresponding relative reciprocal-lattice spacing map for in-plane (a) and out-of-plane (b) planes, and the corresponding relative reciprocal-lattice rotation map for in plane (c) and out-of-plane (d) planes.

Scanning electron diffraction line profiles for 4 nanowires with heights between 34 nm and 219 nm.

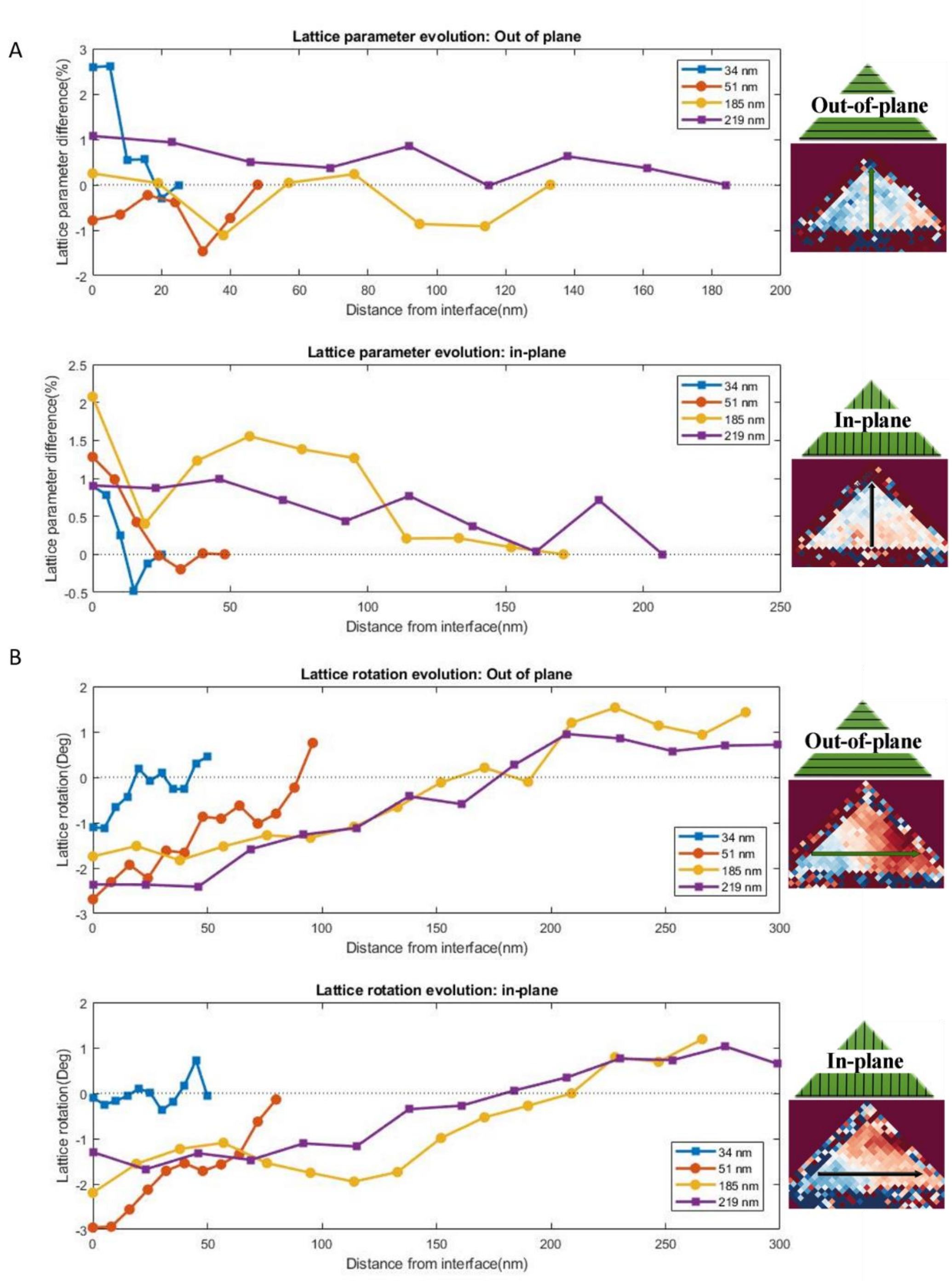

Supplementary Figure 10. Line profiles of (A) the lattice parameter evolution from bottom to apex and (B) lattice rotation across the nanowires extracted from the SED maps in Supplementary Figure 9. Source data are provided as a Source Data file. Source data are provided as a Source Data file.

Additional scanning transmission electron microscopy and geometric phase analysis.

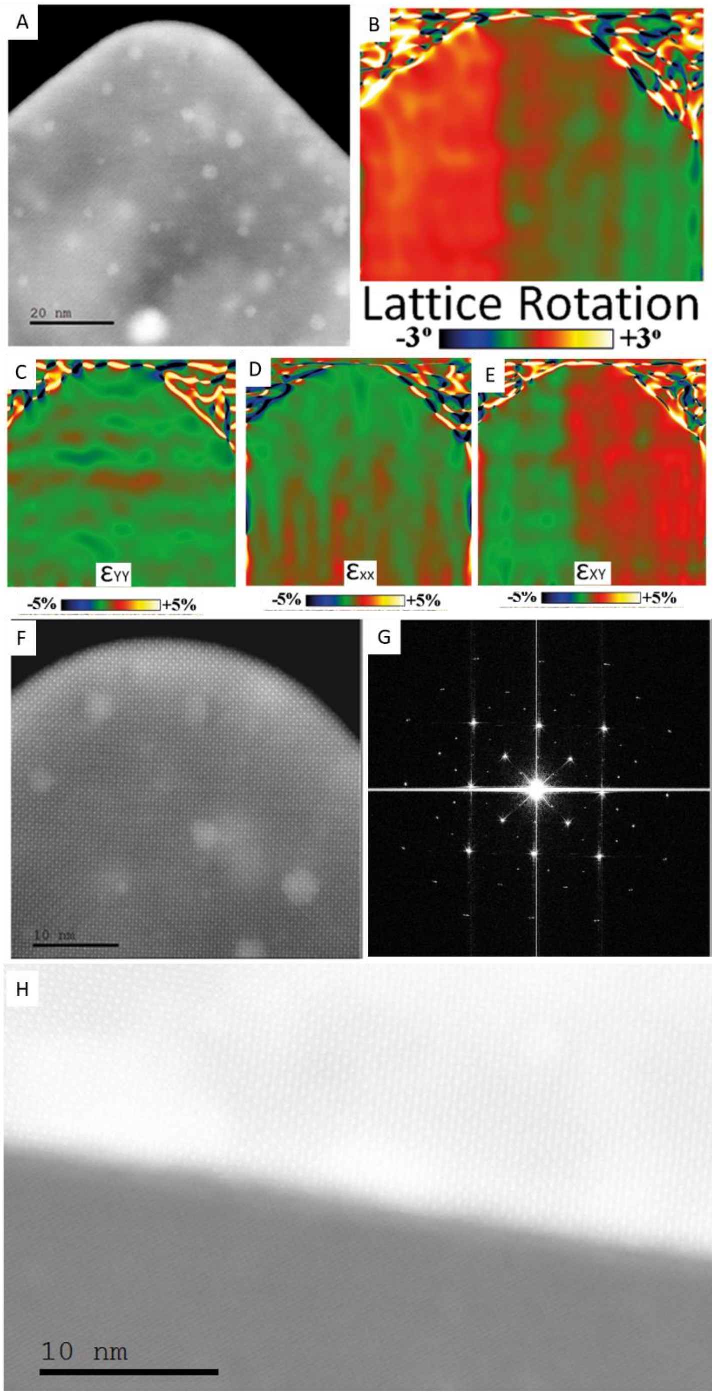

Supplementary Figure 11. Atomic scale imaging and geometric phase analysis (GPA) of surface guided $CsPbBr_3$ nanowires. (A) High-resolution STEM image of the apex of the nanowire. (B) GPA generated lattice rotation map and maps for in-plane (C), out-of-plane (D) and shared (E) strain. (F) Higher magnification image of the apex. (G) Selected area fast Fourier transform of the apex with peaks that correspond to lattice distances that match half a unit cell (H) Closer look at the unresolved interface between the nanowire and sapphire.
